# The dual role of Andean topography in primary divergence: functional and neutral variation among populations of the hummingbird, *Metallura tyrianthina*

**DOI:** 10.1186/s12862-016-0595-2

**Published:** 2016-01-22

**Authors:** Phred M. Benham, Christopher C. Witt

**Affiliations:** Department of Biology and Museum of Southwestern Biology, University of New Mexico, 167 Castetter Hall MSC03 2020, 1 University of New Mexico, Albuquerque, NM 87131 USA; Present address: Division of Biological Sciences, University of Montana, 32 Campus Dr. HS104, Missoula, MT 59812 USA

**Keywords:** Andes, Ecological speciation, Geographic speciation, Bill morphology, Trochilidae

## Abstract

**Background:**

The ridges and valleys of the Andes create physical barriers that limit animal dispersal and cause deterministic local variation in rainfall. This has resulted in physical isolation of animal populations and variation in habitats, each of which has likely contributed to the evolution of high species diversity in the region. However, the relative influences of geographic isolation, ecoclimatic conditions, and their potential interactions remain poorly understood. To address this, we compared patterns of genetic and morphological diversity in Peruvian populations of the hummingbird *Metallura tyrianthina.*

**Results:**

Phylogenetic and variation partitioning analyses showed that geographic isolation rather than climatic dissimilarity explained the greatest proportion of genetic variance. In contrast, bill length variation was explained by climatic seasonality, but not by genetic divergence. We found that mutation-scaled migration rate (*m*) between persistently humid and semi-humid environments was nearly 20 times higher when the habitats were contiguous (*m =* 39.9*)* than when separated by a barrier, the Cordillera de Vilcanota (*m =* 2.1). Moreover, the population experiencing more gene flow exhibited a lesser degree of bill length divergence despite similar differences in climate.

**Conclusions:**

Geographic isolation is necessary for genetic divergence. Ecological differences, represented here by climate characteristics, are necessary for functional divergence. Gene flow appears to hinder the evolution of functional traits toward local adaptive optima. This suggests that functional diversification requires geographic isolation followed or accompanied by a shift in ecological conditions. Andean topography causes both isolation and climatic variation, underscoring its dual role in biotic diversification.

**Electronic supplementary material:**

The online version of this article (doi:10.1186/s12862-016-0595-2) contains supplementary material, which is available to authorized users.

## Background

The tropical Andes harbor a significant fraction of global diversity (e.g., 15 % of land plant species), with half this diversity comprised of regional endemics [1]. In contrast to other diverse montane regions (e.g. the Himalayas [2]), a major fraction of Andean diversity is derived from lineages that have radiated extensively *in situ* [3–9]. Thus, identifying the historical and landscape characteristics that foster speciation in the Andes will be crucial for understanding the mechanisms that have made the region a diversification hotspot. The topographically complex Andean landscape seems to drive diversification in two ways [10]. First, topographic barriers can fragment the narrow distributions of montane taxa and promote divergence via allopatric speciation [11–15]. Second, topography creates climate variation over small spatial scales that can drive ecological divergence, leading to reproductive isolation [16–18] and/or accelerated niche divergence [19]. Although some evidence for both mechanisms operating in the Andes exists, the relative importance of each mechanism working independently or in concert during the diversification process remains poorly understood.

Topographic barriers are frequently associated with phenotypic and genetic discontinuities in Andean species, providing evidence for the importance of physical isolation in Andean diversification (e.g., [20–22]). However, if physical isolation across topographic barriers were the sole driver of Andean speciation, species-level differences would be expected to accumulate via neutral processes. Substantial empirical evidence supports non-neutral mechanisms of speciation [23], although the overall tempo of diversification in the tree of life has recently been interpreted as evidence of neutral diversification [24]. Discordant patterns of phenotypic and genetic diversity are frequently found in Andean lineages, in contrast to neutral expectations ([4, 25–27], but see [22]). Moreover, coalescent simulations of plumage evolution in *Arremon* brushfinches indicate that plumage differentiation proceeds at a much faster rate than expected via neutral divergence [28]. In sum, it seems that range fragmentation alone is an insufficient mechanism for explaining the generation of Andean diversity.

Ecological speciation occurs when ecological factors, regardless of gene flow, catalyze reproductive isolation by natural selection [18]. In the Andes, higher diversification rates are associated with climatic-niche shifts indicating that ecoclimatic variation may be an important driver of lineage proliferation [29]. Several studies of Andean taxa have found morphometric and functional divergence arising among populations distributed along ecological gradients in the face of ongoing gene flow [30–35]. Although these results suggest that ecological mechanisms might be the primary drivers of divergence in the region, abundant evidence from outside the Andes indicates that rates of phenotypic divergence and evolution of reproductive isolation will be slower in populations experiencing gene flow relative to those that are more isolated (e.g., [17, 36–38]). Indeed, studies examining whether sister species replace one another along ecological gradients in the Andes have been inconclusive, with some sister species found to replace one another along ecological gradients in Andean butterflies [6] and frogs [39], but not in Andean birds or mammals [40–44].

Given the lack of clear support for an exclusive role of geographic isolation or ecological speciation in driving Andean diversification, a better understanding of the speciation process in the Andes will likely come from addressing how geographic and ecological isolation operate in concert [12, 15]. The topographic complexity of the region should allow for interactions between these two processes. Topographic barriers promote the reduction of gene flow, while the increased climatic variation associated with topography potentially increases the tempo of divergence via differential selection pressures [19, 45, 46]. One approach to test whether divergence is associated with topographic relief will be to assay divergence among population pairs that (i) occupy similar climates on either side of a topographic barrier, (ii) occupy different climates on either side of a topographic barrier, and (iii) occupy different climates in the absence of a topographic barrier. In particular, comparing patterns of spatial variation in effectively neutral genetic markers and functional traits will provide a means to understand the relative influence of geographic and ecological isolation in promoting genetic structure and divergence in ecologically relevant traits.

**Fig. 1:**
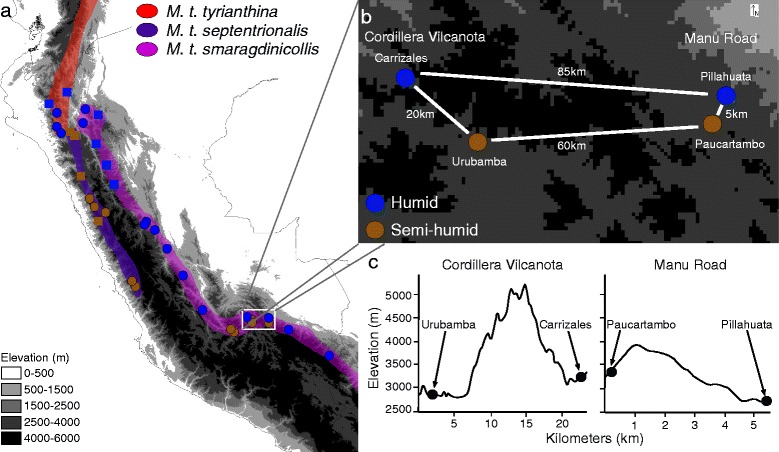
**a** Map illustrating the distribution of all three *Metallura tyrianthina* subspecies that occur in Peru. Circles denote sampling sites for both genetic and bill length data; squares represent sampling sites for bill length data only. Blue points are sites where the habitat was characterized as humid montane forest and brown points semi-humid montane scrub. **b** Map of sampling sites from the department of Cusco, Peru. One pair of sites was from the humid (Carrizales) and drier (Urubamba) sides of the Cordillera de Vilcanota, separated by ~20 km. A second pair of sites was along the Manu Road, with a humid (Pillahuata) and drier (Paucartambo) site ~5 km apart. **c** Topographic profile of the Cordillera de Vilcanota, which exceeds 5000 m, and the Manu Road where a cordillera less than 4000 m in elevation separates the two sites along the Manu Road

We focus on Peruvian populations of the Andean hummingbird *Metallura tyrianthina* to understand the drivers of spatial diversity in functional morphology and genetics. The geological history of the Peruvian Andes generated a topographically complex landscape, with glaciated cordilleras >5000 m in elevation interspersed with deep river valleys [47]. These landscape features generated habitat discontinuities in the elevational distribution of *M. tyrianthina* (1900–4200 m [48]). Final uplift of the central Andes over the last ~10 Myr also dramatically altered the climate [49], generating a steep rainfall gradient from the wet eastern slope to the desert-like western slope [50]. *M. tyrianthina* likely colonized Peru from northern populations ~2 Ma and presently spans several topographic barriers and steep climatic gradients in the Peruvian Andes [51]. Three subspecies of *Metallura tyrianthina* occur in Peru: *M. t. tyrianthina* north and west of the Marañón valley in humid montane forests and edge habitat; *M. t. septentrionalis* in semi-humid montane scrub along the Western Cordillera of Peru south to the department of Lima; and *M. t. smaragdinicollis* throughout the eastern Andes of Peru in humid forests along east-facing slopes and in semi-humid scrub on west-facing and rain-shadowed slopes (Fig. 1a [48]).

We compared spatial patterns of variation in bill length and effectively neutral DNA sequences in *M. tyrianthina* to test predictions derived from models of geographic speciation, ecological speciation, or a combination. (1) If geographic isolation drives divergence then we will expect to find a concordant relationship between phenotypic and genetic divergence across topographic barriers. However, (2) if divergence is primarily driven by ecological factors then we expect to find linked phenotypic and genetic divergence in association with ecological dissimilarities, independent of geographic isolation. Finally, (3) if topographic barriers and ecological differences interact to drive divergence then we expect to find phenotypic divergence between different environments, but only when a topographic barrier that reduces gene flow separates those environments.

## Methods

### Sampling

Tissue samples of *Metallura tyrianthina* were obtained from frozen tissue collections at natural history museums, primarily the Museum of Southwestern Biology (110 individuals). Collection of these samples was approved by the University of New Mexico Institutional Animal Care and Use Committee (protocol no. 08UNM033-TR-100117) and permitted by Peruvian management authorities (permit nos. 76-2006-INRENA-IFFS-DCB, 004-2007-INRENA-IFFS-DCB, 135-2009-AG-DGFFS-DGEFFS, 0377-2010-AG-DGFFS-DGEFFS, 0199-2012-AG-DGFFS-DGEFFS, and 006-2013-MINAGRI-DGFFS/DGEFFS). The geographic breadth of sampling was increased through tissue loans of an additional 26 individuals from other natural history collections (Additional file 1: Table S1). From all 136 individuals, we extracted genomic DNA from pectoral muscle that was either frozen or preserved in RNAlater® (Life Technologies) using a Qiagen DNeasy blood and tissue kit and following the manufacturer’s protocols (Qiagen, Valencia, CA, USA). The mitochondrial gene NADH dehydrogenase, subunit 2 (*ND2*) was sequenced for all 136 individuals. For all of the 47 individuals sampled in the department of Cusco we sequenced an additional two nuclear introns: Adenylate Kinase, intron 5 (*AK1*) and β-fibrinogen, intron 7 (*Bfib7*); and the Z-linked locus Muscle, Skeletal, Tyrosine Receptor Kinase (*MUSK*). Primers and sequencing protocols were identical to those used in Benham et al. [51]. All sequences were manually assembled and edited using Sequencer v. 4.10.1 (Gene Codes Corporation, Ann Arbor, MI, USA) and aligned using MUSCLE v. 3.7 [52]. Haplotype reconstruction for nuclear loci with multiple double peaks was conducted using the program PHASE v. 2.1.1 [53, 54].

The size and shape of hummingbird bills vary in association with floral resources [55, 56] and competitive interactions with other hummingbirds [57]. The hummingbird bill is an ideal trait in which to examine functional diversification across the Andean landscape due to its functional importance and the fact that bill characteristics tend to be highly heritable [58]. We measured bill length (exposed culmen) to the nearest 0.01 mm using digital calipers on 245 museum specimens collected from throughout Peru (Fig. 1a; Additional file 1: Table S1). All specimens included in this study were associated with precise locality and mass data and all measurements were made on dried specimens to avoid increased error from specimen shrinkage [59]. Sexual dimorphism in bill length and shape is widely encountered within Trochilidae [60, 61]; however, using a two-tailed *t*-test for the entire dataset, we did not detect any significant differences between the sexes in bill length; accordingly, males and females were pooled for subsequent analyses. All statistical analyses were conducted using the open source program R (http://www.r-project.org/).

To assess environmental differences among all specimen localities we obtained bioclimatic data at 30-s resolution from the WorldClim dataset [62]. Using coordinates for each sampling locality we extracted data from gridfiles of the BioClim variables within the program DIVA-GIS (http://www.diva-gis.org/). The climatic data includes 19 variables related to measures of temperature, precipitation and seasonality (Additional file 1: Table S2). These data are derived from interpolated climate surfaces available for the entire globe at 30-arc sec spatial resolution and were gathered from several independent sources between 1950 and 2000 [62]. Although the WorldClim dataset may be error-prone in montane regions due to interpolation [62], such error is likely to be random with respect to our hypotheses. The WorldClim dataset has been used successfully in a number of modeling and morphological studies of Andean birds (e.g., [63–65]). WorldClim data has also been used successfully at fine spatial scales to guide surveys for range-restricted Andean hummingbird species [66]. We divided the 19 BioClim variables into three groups each corresponding to variables related to temperature, precipitation, and seasonality. Secondly, we performed a principal components analysis on each of the three groups and used the first components of temperature (83.7 % of the variance), precipitation (78.0 %), and seasonality (82.9 %), respectively, for analyses (Additional file 1: Table S2).

### Influence of topographic barriers on divergence

To assess the association of genetic structure with topographic barriers we constructed a phylogeny of all *Metallura tyrianthina ND2* samples using Bayesian methods in MrBayes v. 3.1 [67] on the CIPRES Science Gateway [68]. For an outgroup we obtained *ND2* data of *Metallura phoebe* from GenBank (Ascension number: EU042569.1). A GTR + I + Γ model was selected as the most appropriate substitution model based on Akaike Information Criteria [69] estimated in jmodeltest v. 0.1 [70, 71]. We also partitioned the dataset by codon position following McGuire et al. [5]. The MCMC analysis ran for 40 million generations sampling every 1000 generations and consisted of four simultaneous runs of four chains each with a temperature for heated chains of 0.175. We assessed convergence using the program AWTY [72] and discarded the first 10 % of trees as burnin. To further evaluate patterns of genetic structure we calculated the number of haplotypes for *ND2* in DNaSP [73] and visualized these as a haplotype network using a median-joining method in NETWORK v. 4.6.10 [74].

**Fig. 2:**
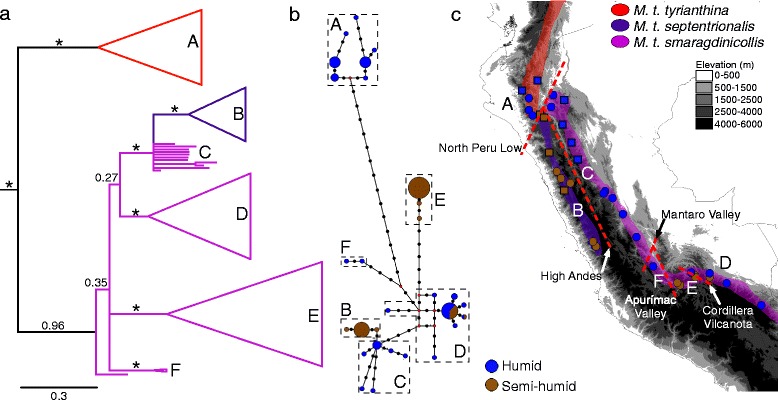
**a** *ND2* Bayesian phylogeny for Peruvian populations of *Metallura tyrianthina.* Posterior probabilities for each node printed above branches (* signifies 1.0 pp). The six geographically structured clades are lettered on all figures. Each clade is colored by subspecies as in panel c. **b** Median-joining haplotype network of *ND2*. Brown color indicates semi-humid montane scrub habitat; blue indicates humid montane forest. **c** Geographic distribution of each clade (A-F). Red dotted lines signify putative physical barriers that isolate the six clades

We evaluated the proportion of genetic diversity explained by topographic barriers as identified with phylogenetic and network analyses in Peruvian *Metallura tyrianthina* using an AMOVA analysis. For this analysis we divided the sampled individuals into six groups each divided by a topographic barrier. These barriers include: the North Peru Low, high Andean ridgeline, the Mantaro valley, the Apurímac valley, and the Cordillera de Vilcanota (Fig. 2c). These barriers also correspond to phenotypic and genetic discontinuities in other Andean bird species (e.g., [20, 75]). Degree of divergence among clades was assessed with Fst values and Nei’s average corrected pairwise differences (D_A_ [76]), which accounts for intra-population polymorphism with the equation D_A_ = d_xy_ - 0.5 (d_x_-d_y_), where x and y are the two populations compared and d is the average uncorrected genetic differences. For each of the clades identified using the above analyses we also calculated standard indices of molecular diversity, including nucleotide and haplotype diversity. Finally, we calculated Tajima’s D and Fu’s F (1000 simulations) to determine whether any of the *ND2* clades exhibit deviations from neutrality. All population genetic analyses were performed in the program Arlequin v. 3.5 [77].

### Concordance between genetic and morphological divergence

To test for concordance between patterns of genetic divergence and bill length variation we compared bill length differences to two measures of genetic divergence, D_A_ and linearized Fst (equal to Fst/1-Fst [78]), across sixteen sampling localities. These measures of genetic distance were compared to the average difference in bill length between each pair of sampling localities. We used a series of linear regression analyses to compare genetic and bill length differences among all pairwise comparisons of populations, among populations in humid montane forest habitats, in semi-humid montane scrub habitats, and among populations distributed in different habitat types (Fig. 1a).

### Climatic drivers of divergence

Populations distributed across different climatic regimes could experience adaptive divergence that will have a negative influence on gene flow leading to reproductive isolation and genetic divergence even at neutral loci, a process referred to as isolation by environment (IBE [18, 79–81]). We test for IBE in *Metallura tyrianthina* by comparing the relative roles of climatic distance and geographic distance in shaping patterns of genetic variation using a variation partitioning method in the R-package ‘vegan’ [82]. Variation partitioning utilizes canonical redundancy analysis to dissect the contributions of different explanatory factors (e.g. climate, geographic distance) to variation in a response variable (e.g., genetic distance [83]). Variation partitioning has been shown to perform similarly to other recently developed tests for teasing apart spatially autocorrelated drivers of genetic diversity [80] and performs better than partial Mantel tests, which can exhibit a high type I error rate when considering spatially autocorrelated data [81, 84, 85]. For *M. tyrianthina* we assessed the influence of environmental variation on genetic distance as measured by D_A_ and linearized Fst. We used Euclidean geographic distances among sampling localities to assess the role of geographic isolation. To assess the role of climate, we performed principal components analysis on all 19 BioClim variables from the 16 sampling sites for which we had both genetic and bill length data to reduce these variables to two principal components. The first principle component explained 93.13 % of BioClim variation, with the heaviest loading linked to annual precipitation. The second principle component explained 4.55 % of BioClim variation, with the heaviest loading associated with precipitation in the wettest quarter (Additional file 1: Table S3). As alternative climatic variables, we used the first principle components of the BioClim variables for temperature, seasonality, and precipitation, respectively. Using either D_A_ or linearized Fst we performed a series of variation partitioning analyses using all potential combinations of climatic principal components plus geographic distance as the explanatory variables (Table 1). We then tested whether the fraction of variation explained by each climatic variable was significant when conditioned for geography, and vice versa, using a permutation analysis with the ‘anova’ function in the vegan package (10,000 permutations).

**Table 1:** Proportion of genetic variance explained by geographic or climatic variation

	D_A_			Fst/(1-Fst)		
Model	Geography	Climate	Shared	Geography	Climate	Shared
geog + [clim1,clim2]	0.4001	−0.0278	0.0660	0.1557	0.0078	0.0969
geog + clim1	0.4838*	0.0102	−0.0176	0.2332	0.0578	0.0194
geog + clim2	0.3392*	−0.0841	0.1270	0.1010	−0.1316	0.1516
geog + [temp,seas,prec]	0.8526***	0.4035*	−0.3864	0.2404	0.1287	0.0122
geog + prec	0.4832*	0.0095	−0.0170	0.2321	0.0569	0.0205
geog + seas	0.4865*	0.054	−0.0204	0.3068	0.0545	−0.0545
geog + temp	0.4917*	−0.0295	−0.0256	0.2047	−0.0928	0.0479
geog + [prec,seas]	0.3636	−0.047	0.1026	0.1007	−0.0349	0.1518
geog + [prec,temp]	0.5093	−0.0238	−0.0432	0.1870	−0.0294	0.0655
geog + [seas,temp]	0.6909***	0.2009	−0.2248	0.3160	0.0240	−0.0635

Geographic distance (geog) is the Euclidean distance among sampling sites and climate is the proportion of variance explained by the different climatic variables included in each model. Clim1 and clim2 are the first two principal components of the entire 19 variable BioClim dataset, explaining 97 % of the variance in total. Temp, seas, and prec are the first principal components for temperature, seasonality, and precipitation BioClim variables, respectively. The proportion of variance shared by both geography and climate is reported in the columns labeled ‘shared’. High ‘shared’ values would indicate collinearity between ecological and geographic parameters; negative values are an artifact of subtracting adjusted R^2^ values to derive the shared proportion of variance [83]

*p < 0.5; **p < 0.01; ***p < 0.001

We tested the influence of different climatic variables on bill length variation throughout Peru using an information-theoretic approach. We performed regression analyses with bill length as the dependent variable and PC1 of temperature, precipitation, and seasonality, respectively, as explanatory variables. We also explored the possibility of using PC1 and PC2 of all 19 BioClim variables as predictors; however, PC1 and PC2 were found to be strongly correlated with seasonality and temperature, respectively (correlation coefficient >0.95 for both). Consequently, we excluded PC1 and PC2 predictors to avoid issues associated with multi-collinearity in downstream analyses. Finally, we also include body mass in this analysis as another potential predictor of bill length variation. We evaluated 15 models representing all possible main effect combinations of predictor variables and included an additional six models to evaluate all possible pairwise interactions between the four variables. The likelihood of all 21 models was evaluated using an Akaike Information Criterion corrected for sample size (AICc). Each model was ranked using ΔAICc and Akaike weights [86] to determine which of our hypothesized climatic models best explains bill length variation in Peru. We discarded models from consideration if a nested model (containing a subset of the same parameters) had a better AICc score [87].

### Dual influence of topography on divergence

We assess the combined influence of topographic barriers and climatic differences in shaping patterns of genetic and morphological divergence across two pairs of populations in which the members of each pair are adjacent but occur in different environments (Fig. 1b). The first pair of populations were 20 km apart on either side of the Cordillera de Vilcanota with one population sampled in the semi-humid scrub of the Urubamba valley (*n* = 21; elevation range 3380–4200 m) and the second within humid montane forest near the town of Carrizales (*n* = 6; elevation: 3201–3552 m). A second pair of populations was distributed along the Manu Road only 5 km apart with birds sampled in the semi-humid scrub near the town of Paucartambo (*n* = 7; elevation 3360 m) and in humid montane forest near Pillahuata (*n* = 13; elevation 2500–3350 m). The Cordillera de Vilcanota is glaciated and exceeds 5000 m elevation, with passes as low as 4300 m; whereas the pass separating humid and semi-humid populations along the Manu Road is only 3900 m (Fig. 1c). Given that the typical elevational distribution of *M. tyrianthina* is 1900–4200 m [46] the Cordillera de Vilcanota likely imposes a more significant barrier to gene flow then any ridge along the Manu Road. We used a one-way ANOVA and TukeyHSD test to examine differences in bill length among the four sites. To determine how our small sample sizes for the Carrizales (*n* = 6) and Paucartambo (*n* = 7) populations would impact our statistical power, we performed a power analysis in the R package pwr (https://cran.r-project.org/web/packages/pwr/index.html). For this analysis we first performed two *t*-tests comparing bill length differences across the Cordillera de Vilcanota and the Manu Road. The results of these t-tests were used to estimate the effect size of bill length divergence between both humid and semi-humid population pairs. Given the calculated effect of habitat on bill length divergence across both population pairs we confirmed that the statistical power using our small sample sizes exceeded the *a priori* defined threshold of 0.8 (i.e., >80 % probability of rejecting the null hypothesis if it is false).

To assess patterns of genetic divergence among sampling localities we calculated all pairwise Fst-values across the four sampled populations for each of the four loci in Arlequin v.3.5 [77]. Secondly, we determined levels of gene flow among the four populations using the program IMa2 [88]. IMa2 is a coalescent genealogy sampler that simultaneously estimates effective population size, migration rates, and divergence times within the framework of an isolation with migration model [89]. The IMa2 program assumes no recombination and before analyses we assessed patterns of recombination in the nuclear loci using the four-gamete test [90] in DnaSp [73]. This test detected recombination in regions of both *AK1* and *Bfib7*. We saved the longest segment of each gene exhibiting no evidence of recombination, leaving segments of 232 bp for *AK1* and 313 bp for *Bfib7*. We conducted pairwise comparisons between humid and semi-humid populations distributed along the Manu Road and across the Cordillera de Vilcanota. Additionally, we ran analyses comparing the two humid populations and the two semi-humid populations. For each locus we specified a mutation rate with error (equal to mutations per locus per year (μ/l/y)) derived from a multi-locus dataset analyzed in *BEAST using both geological and fossil calibrations for hummingbirds [51]. These mutation rates were: *ND2*: 9.5 × 10^−6^ μ/l/y (95 % HPD: 7.5 × 10^−6^, 1.2 × 10^−5^ μ/l/y); *AK1*: 1.0 × 10^−6^ μ/l/y (95 % HPD: 7.0 × 10^−7^, 1.3 × 10^−6^ μ/l/y); *Bfib7*: 1.3 ×10^−6^ μ/l/y (95 % HPD: 8.5 × 10^−7^, 1.6×10^−6^ μ/l/y); and *MUSK*: 9.8 × 10^−7^ μ/l/y (95 % HPD: 6.4 × 10^−7^, 1.2 × 10^−6^ μ/l/y). We applied inheritance scalars to each locus (*ND2*: 0.25; *MUSK*: 0.75; *AK1*: 1.0; *Bfib7*: 1) to account for variation in effective population sizes. Benham et al. [51] hypothesized that *Metallura tyrianthina* colonized southern Peru ~2 Ma (95 % HPD: ~1-3 Ma) based on a time-calibrated phylogeny. We use the upper bound of the 95 % HPD from this previous analysis to set the prior for divergence time in all analyses as *t* = 3 Ma. We constrained the migration rate parameter *m* to be symmetric to reduce the number of parameters, as preliminary analyses estimating asymmetric migration rates were poorly resolved. We also ran several preliminary runs with large, flat priors to estimate migration rate and population size [91]. Based on these preliminary analyses, we set the upper bound of a uniform prior that included the entire posterior distribution of each parameter (Additional file 1: Table S4). Preliminary analyses were unable to resolve *q* for the ancestral population (θ_A_); accordingly, we followed Peters et al. [92] by setting the upper bound of θ_A_ to be greater than the sum of θ for the two sampled populations. For all four comparisons, we ran three independent MCMC analyses to ensure convergence on similar parameter estimates. We also evaluated stationarity of the MCMC analyses by checking whether ESS was >150 for all parameters and examining plots generated by the program for Log [P(G) + P(D|G)] for evidence of long term trends. Runs ran for 10^5^ steps of burnin and at least 10^7^ steps post-burnin, sampling every 100 steps to generate 10^5^ genealogies. For all runs, we employed a geometric heating scheme with 50 heated chains.

## Results

### Sequence characteristics

*ND2* sequences were 1041 bp in length and included 76 informative sites (7.3 %). A 90 bp indel in the *AK1* intron and a 15 bp indel found in the *Bfib7* intron were removed before any analyses were conducted, leaving: 337 bp of *AK1* (four informative sites–1.2 %), 629 bp of *Bfib7* (five informative sites–0.8 %), and 594 bp of *MUSK* (four informative sites–0.7 %). There were no internal stop codons, indels, or anomalous substitution patterns in the *ND2* sequences that might indicate amplification of pseudogenes. However, we excluded one sample (FMNH 433155) because it did not align with other sequences and BLAST returned ambiguous matches with a variety of hummingbird species. Mitochondrial and nuclear intron sequence data have been deposited on GenBank, accession numbers: KU527140-KU527416.

### Geographic isolation

Seven well-supported mitochondrial clades (posterior probability > 0.9) were recovered in the Bayesian phylogeny (Fig. 2a). This structure only corresponds to the populations sampled in Peru and more extensive phylogeographic structure exists within this species in the northern Andes [51]. The nominate subspecies consists of one clade (A) isolated by the North Peru Low and >2 % divergent from all other populations (Additional file 1: Table S5). Topographic barriers were associated with phylogenetic structure among all other clades, including: *M. t. septentrionalis*, on the west side of the central Andes (clade B), which is 0.24 % divergent from a northern clade of *M. t. smaragdinicollis* on the east side of the central Andes (clade C); clade D of southeastern Peru, which is isolated from clade E (0.91 % divergent), of the upper Apurímac and Urubamba valleys, by the Cordillera de Vilcanota; and finally, an Ayacucho population (clade F) that is bounded by the Mantaro River to the north (0.67 % divergent) and the Aprurímac River to the south (0.79-1.04 % divergent). Some structure was also detected within clade C potentially due to the influence of the Huallaga Valley or isolation by distance. Fst values largely corresponded to corrected pairwise differences among clades (Additional file 1: Table S5). A single individual from the same locality as clade D showed closer affinities to an extra-limital haplogroup from Bolivia (see [51]). Haplotype networks reaffirmed the prominent role of physical barriers in structuring neutral genetic diversity. Haplotype structure between the humid montane forest and semi-humid montane scrub habitats only existed in conjunction with physical barriers. Within clade D, birds from both humid and semi-humid habitats shared identical mtDNA haplotypes (Fig. 2b). Finally, using an AMOVA analysis we found that 88.1 % (d.f. 6; sum of squares 735.2; variance 6.8) of the genetic variance within Peruvian *M. tyrianthina* corresponds to the barriers highlighted in red (Fig. 2c) and only 9.4 % (d.f. 120; sum of squares 86.7; variance 7.2) of the genetic variation is found within groups between topographic barriers (Additional file 1: Table S6). Nucleotide diversity was highest in clades A and C. Only clade D exhibited significant deviations from neutrality based on both Fu’s F and Tajima’s D. Clade B also exhibited significant Fu’s F and clades A and C exhibited significant Tajima’s D (Additional file 1: Table S7). Deviations from neutrality in these clades may reflect the recent expansion of the species into the central Andes [51] or subtle geographic structure among sampling localities within these clades.

### Ecological divergence

Results of variation partitioning analyses corroborated a strong role for isolation by geographic distance, but not ecological isolation, in shaping patterns of genetic variation in Peruvian *Metallura tyrianthina*. When D_A_ was used as a metric for genetic distance, 33.9–85.3 % of the variance was explained by geographic isolation alone compared to the 0.9–40.3 % attributable to certain climatic dissimilarities among localities. In no instance did climatic variation explain patterns of genetic variation better than geographic distance (Table 1). Moreover, the genetic variance explained by geographic distance alone was found to be significant when each of the different climatic models were accounted for using permutation tests. The highest level of genetic variance explained by climate was when PC1 of temperature, precipitation, and seasonality were all included in a model (40.3 %). This was also the only combination of climatic variables found to explain a significant proportion of the genetic variance using the permutation test; however, there was also a high degree of collinearity between geography and climate when all climate parameters were included in the model (shared proportion of variance: −0.386). Patterns for linearized Fst values were consistent with those found for D_A_, in that geographic isolation always explained a greater proportion of the variance (10.07–31.60 %) than climate (0.78–12.87 %). However, less of the overall variance was explained by geographic isolation and, using the same permutation test, none of the variance explained by geographic distance was found to be significant when climate was accounted for (and vice versa).

**Fig. 3:**
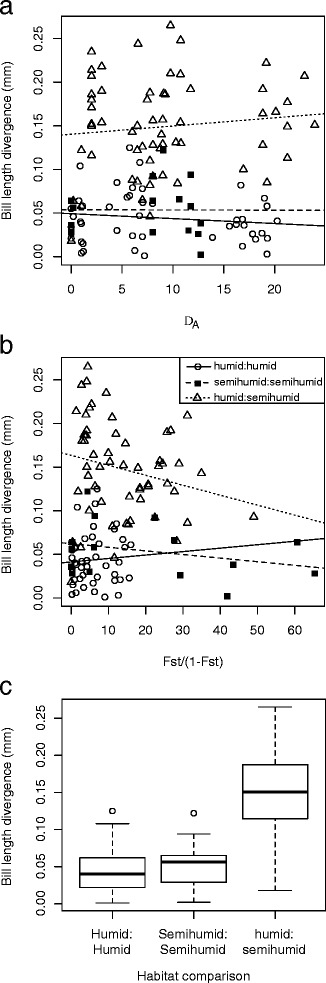
Linear regression of bill length versus **a** pairwise genetic differences (D_A_) and **b** linearized Fst among all populations. No significant relationships were found across all comparisons. **c** Boxplot of mean divergence in bill length between all humid sites, semi-humid sites, and among sites in different habitats. Mean divergence between habitats was significantly greater than that between habitats, whereas within habitat divergence did not differ

A simple linear regression of bill length versus body mass indicated a weak, positive relationship (*p* = 0.015; R^2^ = 0.024); however, body mass was also found to contribute very little to bill length variation in *M. tyrianthina* relative to climatic factors in an AICc analysis (Table 2). Given the weak relationship between body mass and bill length we use length measurements uncorrected for variation in body mass for all subsequent analyses. Divergence in mean bill length was greater in comparisons between habitats than comparisons within habitats (TukeyHSD test; *p* < 0.001; Fig. 3). Bill length divergence was comparable among humid and semi-humid population comparisons, respectively (*p* > 0.1; Fig. 3c). There were no relationships between genetic distance and bill length divergence, regardless of whether we compared all populations, populations within the same habitat, or populations between habitats (for all *p* > 0.1; Fig. 3).

**Table 2:** The two best models predicting bill length variation based on AICc analysis: (1) seasonality, precipitation and their interaction; and (2) seasonality alone

Model	logL	k	AICc	ΔAICc	Model weights
[seas][prec][seas*prec]	−310.02	5	630.29	0	0.7776
[seas]	−313.34	3	632.79	2.50	0.2224
[mass]	−379.04	3	764.18	133.89	0

The 21 models we evaluated include all 15 possible combinations of body mass, PC1 of seasonality (seas), PC1 of precipitation (prec), PC1 of temperature (temp) as well as all six pairwise interactions among the four predictor variables. Relative variable importance (the sum of model weights in which a particular variable appears) was much greater for seasonality (1.0) than precipitation (0.55), temperature (0.35), or body mass (0.37)

**Fig. 4:**
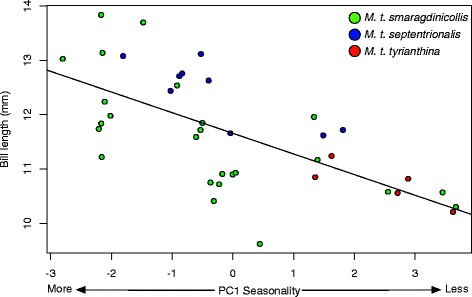
Bill length as a function of PC1 of seasonality (82.9 % of the variation). The linear regression model was highly significant (*p* < 0.0001) with an R^2^ of 0.44

The model including an interaction between PC1 of seasonality and PC1 of precipitation, plus their main effects, was the single best model explaining bill length variation, containing 77.8 % of the Akaike weight. The second best model consisted solely of the PC1 of seasonality (22.2 % Akaike weight; ΔAICc =2.50). Although a number of models were competitively ranked with PC1 of seasonality (i.e., ΔAICc < 2) all of these models include PC1 of seasonality as a nested component and were thus considered uninformative (Table 2; [87]). We additionally quantified the relative importance of each predictor variable by adding together the model weights for each model in which the variable appeared [93]. This analysis highlighted the overwhelming influence of seasonality (1.0) relative to precipitation (0.55), temperature (0.35), or body mass (0.37). Linear regression of bill length versus PC1 of seasonality was also highly significant (*p* < 0.0001; R^2^ = 0.44; Fig. 4). Seasonality remained a significant explanatory variable of bill length variation after accounting for spatial autocorrelation by incorporating data on latitude and longitude from each sampling site into a non-linear mixed effects model in the R package nlme (https://cran.r-project.org/web/packages/nlme/index.html).

### Dual influence of topography on divergence

*ND2, AK1* and *MUSK* all exhibited significant divergence in Fst across the Cordillera de Vilcanota between Urubamba and Carrizales, Fst for *Bfib7* was non-significant (Table 3). Significant Fst structure along the Manu Road was only found for the *AK1* intron, which also had a lower Fst value (0.184) than across the Cordillera de Vilcanota (0.336). Significant genetic structure was also found between the two humid sites (Carrizales and Pillahuata) across all loci and for *ND2* and *AK1* between the two drier sites (Urubamba and Paucartambo).

**Table 3:** Fst-values among pairwise comparisons of all four Cusco localities (Fig. 1b)

	Vilcanota	Manu Road	Humid	Semi-humid
*ND2*	0.802***	−0.051^ns^	0.167*	0.814***
*AK1*	0.336***	0.184*	0.290***	0.245***
*Bfib7*	−0.055^ns^	−0.0387^ns^	0.281*	−0.012^ns^
*MUSK*	0.368***	−0.055^ns^	0.229*	−0.069^ns^

Vilcanota and Manu Road columns each represent a comparison between a pair of humid and semi-humid environments. Humid comparisons are between Carrizales and Pillahuata populations, semi-humid comparisons are between Urubamba and Paucartambo populations

^ns^ *p* > 0.05; *p < 0.05; **p < 0.01; ***p < 0.001

**Fig. 5:**
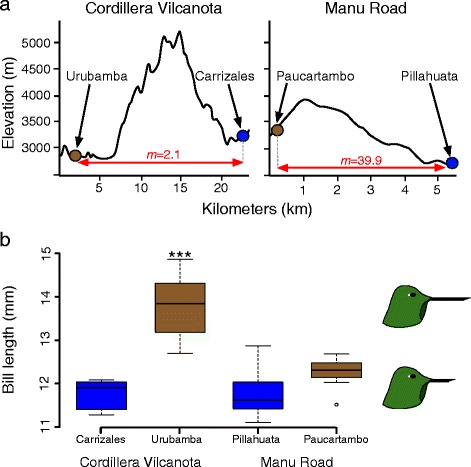
**a** Topographic profiles of the two cordilleras separating sampling sites in the Vilcanota region and along the Manu Road (see Fig. 1c). Arrows with red lettering indicate mean migration rates (*m*) estimated from IMa2 analyses (Table 4). **b** Bill length differences among the four Cusco localities. Blue represents the two humid sites and brown the two drier sites. Asterisks indicate p-values for the comparisons of bill length between two pairs of adjacent populations in humid and semi-humid environments (* < 0.05; ** < 0.001; *** < 0.0001)

IMa2 analyses of populations separated by the Cordillera de Vilcanota and between the two semi-humid sampling localities showed good evidence of convergence with ESS values >150 and all three runs exhibiting similar values for all parameters. By contrast, analyses of the populations along the Manu Road and comparisons between the two humid-site populations did not exhibit evidence for convergence (ESS values <100) after sampling 250,000 genealogies despite similar results being obtained across each of the three independent runs. Migration rates (*m*) based on all four loci across both humid to semi-humid comparisons were found to be greater than zero (Fig. 5a; Table 4); however, average migration rates along the Manu Road (39.92; 95 % HPD: 6.31–150) were estimated to be over 19 times greater than the average migration rate across the Cordillera de Vilcanota (2.10; 95 % HPD: 0.38–9.76). Within habitat migration rates were greater on average between the two humid sites (39.70; 95 % HPD: 1.51–100) than between the two semi-humid sites (3.38; 95 % HPD: 0.68–36.50). Estimated average divergence times were greater between the Urubamba population and all others (0.74–0.94 Ma) than they were between the two Manu Road populations (0.03 Ma) or between the two humid-climate populations (0.03 Ma; Table 4). However, the 95 % HPDs of all divergence time estimates overlapped and resolution of this parameter was generally poor, with a peak found in the distribution of *t* followed by either a long plateau or the values of *t* decreasing in probability but plateauing at a low non-zero value. The poor resolution of dates was most likely due to insufficient data. θ was generally low, varying from 0.14 to 1.40 in all well-resolved populations (Table 4). The posterior distribution of θ_A_ was always poorly resolved as was θ for the population sampled at Pillahuata with estimates exhibiting long non-zero tails within the specified prior distribution. Again, we think that this is due to insufficient data to resolve these estimates rather than an inappropriate designation of the upper bound of the prior given that all other population sizes were much smaller than the upper bound (see above).

**Table 4:** Parameter estimates for θ (4N_e_μ), divergence time (*t*), and symmetrical migration rates (*m*) inferred from IMa2 analyses

Transect	θ_1_	θ_2_	θ _ancestral_	*t*	*m*
Vilcanota	0.321 (0.103–0.903)	0.807 (0.205–3.117)	0.06 (0.00–4.00)	0.7985 (0.295–3.0)	2.098 (0.383–9.759)
Manu Road	1.402 (0.027–0.379)	4.00 (0.140–4.00)	1.521 (0.00–4.00)	0.031 (0.016–2.998)	39.922 (6.308–150)
Humid	6.000 (0.042–6.0)	0.140 (0.036–0.499)	0.587 (0–6.00)	0.026 (0.001–2.998)	39.702 (1.513–100)
Semi-humid	0.260 (0.062–0.973)	0.77 (0.039–1.199)	1.746 (0.0–4.00)	0.939 (0.052–2.998)	3.379 (0.676–36.501)

95 % HPD range for each parameter presented in parentheses. Mean parameter estimates represent the average across the three independent runs performed for all four comparisons. θ_1_ refers to estimates of θ for the semi-humid comparison for Vilcanota and Manu Road and θ_2_ for the humid site. For between humid sites and semi-humid site comparisons θ_1_ refers to sites from the Manu Road and θ_2_ the Vilcanota

Cusco populations on the drier, west-facing slopes had longer bills than those on the humid, east-facing slopes (Fig. 5b). Bill length in Urubamba was significantly longer than all three of the other populations (*p* < 0.0001). Although bill length in Paucartambo was slightly longer (average 0.47 mm) than birds from the adjacent humid slopes at Pillahuata, this result was not significant when corrected for multiple comparisons (*p* = 0.216). There were no differences in bill length between the two humid sites (*p* > 0.4).

## Discussion

### Geographic isolation promotes genetic divergence

Phylogenetic, network, and AMOVA analyses all point to an important role for topographic barriers in structuring genetic diversity in *Metallura tyrianthina*, a result consistent with studies of the Genus *Metallura* as a whole [51, 94] and many other Andean taxa (e.g. [9, 21, 40, 95–97]). We found the North Peru Low, Apurímac Valley and high Andean ridges to be associated with genetic structure in *M. tyrianthina* as in many other taxa [20, 97, 98]. We also found the Cordillera de Vilcanota to be an important landscape feature structuring genetic and phenotypic diversity; the same cordillera was linked to an older phenotypic and genetic divergence in the hummingbird *Adelomyia melanogenys* [65]. Topographic barriers were implicated in generating neutral genetic divergence, but not divergence in bill length (Fig. 3; [22])*.* This suggests that hummingbird bill morphology does not evolve steadily over time, but diverges only in response to differential ecological pressures.

Although we found discordant patterns of genetic and morphological divergence in *M. tyrianthina*, patterns of plumage variation in the species appear to vary in association with topographic barriers and the degree of genetic divergence. A high Andean ridge separates the similar looking subspecies *M. t. smaragdinicollis* and *M. t. septentrionalis,* and the Marañón valley corresponds to turnover between the red-tailed subspecies, *M. t. tyrianthina,* and the two purple-tailed subspecies occupying the rest of Peru [48]. Plumage variation among these subspecies cannot be explained by the climatic characteristics of the regions in which they are distributed. Instead, plumage divergence generally corresponds with time since divergence. The two similar purple-tailed subspecies, which occupy different environments, were found to be only 0.24 % divergent at the *ND2* locus; whereas divergence between red and purple-tailed subspecies distributed in similar environments across the Marañón valley was ~2.2 %. Concordance between tail color and genetic divergence in *M. tyrianthina* is consistent with the positive correlation between the extent of plumage and mtDNA divergence across the Marañón valley, based on multiple passerine genera [22]. Plumage divergence rate appears to be linked to neutral genetic divergence and decoupled from functional trait divergence (but see [28]).

### Bill length varies in association with climate

Populations of *Metallura tyrianthina* occupying habitats characterized by semi-humid montane scrub were found to have significantly longer bills than birds in humid forest habitats (Fig. 3c). This marked morphological divergence was not associated with neutral genetic divergence; rather, we found a strong relationship between climatic and bill length variation, with a model of seasonality, precipitation and their interaction being the strongest predictor of bill variation (Table 1) and seasonality being the most important single predictor (Fig. 4). Our estimate of the strength of this relationship (r^2^ = 0.44) is likely conservative considering that the climate data is coarsely interpolated from unevenly distributed weather stations in the Peruvian Andes. In the Andes, consistent patterns of morphological divergence occurring in association with ecological dissimilarities have been found in *Adelomyia* hummingbirds [26, 65] and *Glyphorhynchus* woodcreepers [32, 64]. A similar relationship between bill length and climate was found in the hummingbird *Adelomyia melanogenys,* where 57 % of the variance in bill length was explained by precipitation seasonality, minimum temperature of the coldest month, and mean elevation [65]. These results suggest that climatic variables or other ecological factors correlated with climate (see below) may play important roles in shaping the impressive diversity of hummingbird bill morphology in the Andes. Concordance between ecological variation and morphological variation is well documented in organisms [99] especially birds where bill morphology responds readily to climatic and dietary change [100–102]. Patterns of bill morphology in *M. tyrianthina* contribute to mounting evidence that the ecological differences generated by the topographically complex Andean landscape play an important role in promoting local adaptation and functional diversity in Andean taxa [28, 30, 32, 33, 65].

The variation partitioning approach we used to test for IBE revealed that geographic distance, not climatic variation, explained the greatest proportion of genetic variance. These results provide additional evidence for an exclusive role of geographic isolation in promoting genetic divergence at the neutral loci studied. Our results resemble a similar study in Trinidadian guppies where no influence of ecological dissimilarity on genetic variation was detected [103]. However, both a study of Caribbean *Anolis* species [80] and a meta-analysis [79] found consistent roles for ecologically mediated genetic divergence when comparing the influence of geographic and ecological distance on patterns of genetic variation. In the Andes, similar analyses assaying genetic variation among populations of the woodcreeper *Glyphorynchus spirurus* in Ecuador found that the Andean ridge separating eastern and western populations played the largest role in structuring genetic divergence. Whereas genetic variance within eastern or western populations was explained largely by ecological variation [64]. Although we do not find evidence for isolation by environment in *M. tyrianthina*, negative results for this test can be difficult to interpret due to factors such as insufficient time for divergence to occur at neutral loci or inappropriate ecological variables selected [18]. Other distance estimates, such as resistance-based distances [104], consistently explain a greater proportion of the variance in genetic diversity than Euclidean geographic distance [80]. Thus, our use of Euclidean distances likely resulted in a conservative estimate of the effect of geographic features on genetic divergence. In general, the results of our variation partitioning and phylogeographic analyses implicate geographic isolation as the major driver of neutral genetic divergence in this species, with little or no additional divergence attributable to ecological differences.

### Barriers facilitate functional divergence

In a wide array of taxa, mismatches between morphology and neutral genetic diversity have been interpreted as evidence for phenotypic divergence with gene flow [26, 32, 105–108]. These studies have generally compared morphology of adjacent populations in different environments versus isolated populations in similar environments; however, they do not assess the extent to which gene flow might prevent populations from achieving local adaptive optima. We compared patterns of gene flow and morphological divergence between pairs of populations in humid and semi-humid environments that were either isolated by a topographic barrier (Cordillera de Vilcanota) or not (Manu Road; Fig. 5a). This allowed us to assess how interactions between barriers and climate might facilitate functional diversification. At all four loci, we found greater Fst-values across the Cordillera de Vilcanota than the Manu Road, with only a single locus exhibiting an Fst significantly different from zero along the Manu Road (Table 3). Coalescent-based analyses in IMa2 confirmed that migration rates across the Manu Road were significantly greater than across the Cordillera de Vilcanota (Fig. 5a; Table 4). Furthermore, migration rates along the Manu Road were found to be equal to or greater than rates between the two humid sites or between the two semi-humid sites. Although MCMC searches in some IMa2 analyses exhibited uncertain convergence, three independent runs always indicated higher migration rates along the Manu Road than across the Vilcanota, and Fst values corroborated patterns suggested by IMa2 analysis.

We found an inverse relationship between bill length divergence and gene flow, with greater divergence in bill length found across the Cordillera de Vilcanota than the Manu Road (Fig. 5b). A similar relationship between gene flow and adaptive divergence has been found in numerous non-Andean taxa [17, 36–38, 109]. Although this pattern has frequently been interpreted as the result of gene flow constraining adaptive divergence, adaptive divergence could also restrict gene flow to the extent that it reinforces reproductive isolation. Teasing apart these mechanisms is a widely recognized challenge [17, 110]. In the present case, the fact that landscape features appear to explain levels of gene flow (Fig. 5a) suggests that adaptive divergence is constrained by gene flow, rather than the reverse.

An alternative explanation for differences in bill length between Urubamba and Paucartambo is that they reflect differences in the amount of time spent in semi-humid environments. In support of this possibility, migration-rate estimates from IMa2 can be spuriously high, according to two recent simulation studies [111, 112]. Both studies found that in cases of recent divergence (i.e. low Fst), datasets with few loci exhibited high false-positive rates for non-zero migration. Given that we only sequenced four unlinked loci, it is possible that this bias could have conflated the migration rates that we found along the Manu Road. However, we think it is likely that low Fst between populations distributed along the Manu Road was due, at least in part, to ongoing gene flow. These populations are only 5 km apart and the intervening area contains a gradual habitat transition from semi-humid scrub to humid forest. Secondly, the uplift of the Cordillera dividing the two sampled populations along the Manu Road occurred >4 Ma before *M. tyrianthina* colonized the central Andes from the north [45, 51, 113]. Although we consider high gene flow to be the most likely interpretation of low genetic divergence across the Manu Road, our relative estimates of gene flow and divergence time should be interpreted with caution. The most conservative interpretation of our results is that bill length similarity is associated with genetic similarity at the population level, regardless of whether that similarity is due to recent divergence or ongoing connectivity.

Although subtle morphological divergence in the face of gene flow has been demonstrated in other Andean birds (e.g. *Glyphorynchus spirurus* [32]), phylogenetic analyses have failed to find a pattern of sister species replacement along ecological gradients [42, 43, 51]. Our results potentially reconcile these contradictory patterns as we find that adaptive divergence with gene flow is possible, but severely constrained. In Andean birds, it appears that allopatry is a pre-requisite or co-requisite for functional diversification and likely the completion of speciation.

### Selection for longer bills

Repeated evolution of longer bills in drier, more seasonal habitats suggests adaptation by natural selection, although the mechanism by which increased bill length may increase fitness in these environments has yet to be tested. Given that climatic variation likely covaries with a number of other potential ecological pressures, pinpointing the mechanistic basis of inter-population variation in bill length would require rigorous field observations and experimentation. Climate could play a direct role in driving bill length differences; however, bill length variation in *M. tyrianthina* and other *Metallura* species [114] directly contradicts Allen’s Rule [100]. Additionally, even though increased bill size can be a key mechanism for shedding excess heat while conserving water in drier environments [102, 115, 116] hummingbirds acquire large volumes of water from their nectarivorous diets and as a consequence do not suffer from water limitations to the same degree as other birds [117]. Instead, biotic interactions represent more likely drivers of geographic variation in bill length in *Metallura tyrianthina*. First, longer bills in drier, less diverse environments could be caused by competitive release, analogous to bird populations on depauperate islands which tend to exhibit divergent bill morphologies as part of a more generalist phenotype [118–120]. *M. tyrianthina* has a shorter bill than most other hummingbird species in the humid forests of east-facing Andean slopes. Longer bills might allow *M. tyrianthina* to exploit a greater variety of flowers on rain-shadowed slopes that harbor fewer competing nectarivore species. Second, diversifying selection on bill length could be exerted by flower species that have different corolla shapes, a co-evolutionary process that has been well-documented in other hummingbird species [55–57, 121]; this is plausible for *M. tyrianthina* because striking floral species turnover coincides with the climatic gradients along which it occurs.

## Conclusions

Andean ridges and valleys hinder connectivity among populations and also create local climatic variation that imposes differential selection pressures [10, 46]. We investigated this dual role of topography for its effects on diversity in a widespread hummingbird species, *Metallura tyrianthina.* Across the Peruvian distribution of this species, we found that topographic barriers play the dominant role in structuring effectively neutral genetic diversity, whereas climatic variation shapes patterns of bill morphology. Moreover, analyses across four sites in the department of Cusco indicated that topography promotes genetic structure even across small spatial scales (<20 km) and bills are longer on rain-shadowed, west-facing slopes. This fine-scale analysis also revealed how gene flow constrains functional divergence, suggesting that topography and climatic variation interact to promote Andean diversification. If functional divergence driven by climatic variation permits coexistence upon secondary contact [19], it could potentially contribute to the evolution of reproductive isolation at later stages of divergence [18]. These processes could increase the overall tempo of diversification [122, 123]. Based on our results from *Metallura*, the east-to-west precipitation gradient may have played an important role in the diversification of several avian genera that contain narrowly distributed and geographically variable taxa in the central Andes (e.g. *Ochthoeca, Anairetes, Atlapetes, Cranioleuca, Spinus*). Indeed, analyses comparing speciation rates among Andean and adjacent lowland lineages have found overall higher speciation rates in the Andes, a pattern driven in part by genera such as *Cranioleuca* [124, 125] and *Spinus* [125, 126]. Topographic barriers and ecological variation have clearly interacted to produce high species diversification rates in Andean birds. The current results suggest that rates of functional diversification should also be accelerated when physical landscape features coincide with ecoclimatic variation.

## Availability of supporting data

The bill length dataset supporting the results of this article are included within the article (and its additional file(s)). Mitochondrial and nuclear intron sequence data have been deposited on GenBank, accession numbers: KU527140-KU527416.

## Additional file

Additional file 1: Table S1. Museum specimens and tissues used in study, sampling localities, bill length measurements, and body mass. **Table S2.** Loadings from principal component analysis of temperature, precipitation, and seasonality Bioclim variables. **Table S3.** Loadings from principal components analysis of all 19 BioClim variables. **Table S4.** Parameter settings for each of the four IMa2 analyses. **Table S5.** Corrected pairwise differences and Fst values among all populations. **Table S6.** Results of AMOVA analysis. **Table S7.** Standard indices of molecular diversity for each clade based on *ND2*. (DOCX 174 kb)
