# Supplementary material for: The dual role of Andean topography in primary divergence: functional and neutral variation among populations of the hummingbird, Metallura tyrianthina
Source: BMC Evol Biol. 2016 Jan 22;16:22. doi: 10.1186/s12862-016-0595-2 (PMC4724075; doi:10.1186/s12862-016-0595-2)
Supplement: Additional file 1: Table S1. — Museum specimens and tissues used in study, sampling localities, bill length measurements, and body mass. Table S2. Loadings from principal component analysis of temperature, precipitation, and seasonality Bioclim variables. Table S3. Loadings from principal components analysis of all 19 BioClim variables. Table S4. Parameter settings for each of the four IMa2 analyses. Table S5. Corrected pairwise differences and Fst values among all populations. Table S6. Results of AMOVA analysis. Table S7. Standard indices of molecular diversity for each clade based on ND2. (DOCX 174 kb) [file 12862_2016_595_MOESM1_ESM.docx]

**Additional file 1**

**The dual role of Andean topography in primary divergence: functional and neutral variation among populations of the hummingbird, *Metallura tyrianthina.***

Phred M. Benham and Christopher C. Witt

**Table S1:** *Metallura tyrianthina* museum specimens and tissue samples used for morphological measurements and sequence data. Measurements of bill length and body mass in last two columns. Individuals without these data were sequenced only. Asterisks mark individuals for which the mtDNA gene ND2 was sequenced for this study and double asterisks refer to the 47 individuals for which both mtDNA and nuclear loci were sequenced. Museum abbreviations are as follows: Louisiana State University Museum of Natural Science (LSUMNZ); Field Museum of Natural History (FMNH); University of Kansas Museum of Natural History (KUMNH); Museum of Southwestern Biology (MSB); Centro de Ornitologia y Biodiversidad (CORBIDI).

| **Voucher #** | **Species** | **Locality** | **Coordinates** | **Bill Length (mm)** | **Body Mass (g)** |
| --- | --- | --- | --- | --- | --- |
| [MSB:BIRD:34998](http://arctos.database.museum/guid/MSB:Bird:34998)* | *septentrionalis* | Ancash, Prov. Caraz; 14.0km SW Caraz | 9.101ºS; 77.865ºW | 11.86 | 3.61 |
| [MSB:BIRD:35026](http://arctos.database.museum/guid/MSB:Bird:35026)* | *septentrionalis* | Ancash, Prov. Caraz; 14.0km SW Caraz | 9.101ºS; 77.865ºW | 13.06 | 3.79 |
| [MSB:BIRD:34928](http://arctos.database.museum/guid/MSB:Bird:34928)* | *septentrionalis* | Ancash, Prov. Carhuaz; 16.0km SE Carhuaz | 9.343ºS; 77.508ºW | 12.65 | 3.85 |
| [MSB:BIRD:34930](http://arctos.database.museum/guid/MSB:Bird:34930)* | *septentrionalis* | Ancash, Prov. Carhuaz; 16.0km SE Carhuaz | 9.343ºS; 77.508ºW | 13.33 | 4.21 |
| [MSB:BIRD:34939](http://arctos.database.museum/guid/MSB:Bird:34939)* | *septentrionalis* | Ancash, Prov. Carhuaz; 16.0km SE Carhuaz | 9.343ºS; 77.508ºW | 12.92 | 3.47 |
| [MSB:BIRD:34944](http://arctos.database.museum/guid/MSB:Bird:34944)* | *septentrionalis* | Ancash, Prov. Carhuaz; 16.0km SE Carhuaz | 9.343ºS; 77.508ºW | 13.16 | 3.96 |
| [MSB:BIRD:34951](http://arctos.database.museum/guid/MSB:Bird:34951)* | *septentrionalis* | Ancash, Prov. Carhuaz; 16.0km SE Carhuaz | 9.343ºS; 77.508ºW | 13.12 | 4.01 |
| [MSB:BIRD:34970](http://arctos.database.museum/guid/MSB:Bird:34970)* | *septentrionalis* | Ancash, Prov. Carhuaz; 16.0km SE Carhuaz | 9.343ºS; 77.508ºW | 13.18 | 3.27 |
| [MSB:BIRD:34988](http://arctos.database.museum/guid/MSB:Bird:34988)* | *septentrionalis* | Ancash, Prov. Carhuaz; 16.0km SE Carhuaz | 9.343ºS; 77.508ºW | 12.86 | 3.91 |
| [MSB:BIRD:34992](http://arctos.database.museum/guid/MSB:Bird:34992)* | *septentrionalis* | Ancash, Prov. Carhuaz; 16.0km SE Carhuaz | 9.343ºS; 77.508ºW | 13.94 | 3.73 |
| [MSB:BIRD:34993](http://arctos.database.museum/guid/MSB:Bird:34993)* | *septentrionalis* | Ancash, Prov. Carhuaz; 16.0km SE Carhuaz | 9.343ºS; 77.508ºW | 13.49 | 3.24 |
| [MSB:BIRD:34984](http://arctos.database.museum/guid/MSB:Bird:34984) | *septentrionalis* | Ancash, Prov. Carhuaz; 16.0km SE Carhuaz | 9.343ºS; 77.508ºW | 12.59 | 3.85 |
| [MSB:BIRD:34815](http://arctos.database.museum/guid/MSB:Bird:34815)* | *septentrionalis* | Ancash, Prov. Santa; Macate | 8.754ºS; 78.048ºW | 11.38 | 3.1 |
| [MSB:BIRD:34817](http://arctos.database.museum/guid/MSB:Bird:34817)* | *septentrionalis* | Ancash, Prov. Santa; Macate | 8.754ºS; 78.048ºW | 12.20 | 3.52 |
| [MSB:BIRD:34822](http://arctos.database.museum/guid/MSB:Bird:34822)* | *septentrionalis* | Ancash, Prov. Santa; Macate | 8.754ºS; 78.048ºW | 13.80 | 4.17 |
| [MSB:BIRD:34823](http://arctos.database.museum/guid/MSB:Bird:34823)* | *septentrionalis* | Ancash, Prov. Santa; Macate | 8.754ºS; 78.048ºW | 12.81 | 3.82 |
| [MSB:BIRD:34827](http://arctos.database.museum/guid/MSB:Bird:34827)* | *septentrionalis* | Ancash, Prov. Santa; Macate | 8.754ºS; 78.048ºW | 13.00 | 3.66 |
| [MSB:BIRD:34866](http://arctos.database.museum/guid/MSB:Bird:34866)* | *septentrionalis* | Ancash, Prov. Santa; Macate | 8.754ºS; 78.048ºW | 13.05 | 3.84 |
| [MSB:BIRD:36046](http://arctos.database.museum/guid/MSB:Bird:36046)* | *septentrionalis* | Ancash; Prov. Yungay, Dist. Yanama, Yanama | 9.018ºS; 77.539ºW |  |  |
| LSUMZ-86026 | *septentrionalis* | Ancash; 31 km (by road) E Paraicoto | 9.550ºS; 77.791ºW | 12.41 | 3.9 |
| LSUMZ-86027 | *septentrionalis* | Ancash; 31 km (by road) E Paraicoto | 9.550ºS; 77.791ºW | 12.86 | 3.8 |
| LSUMZ-86029 | *septentrionalis* | Ancash; 31 km (by road) E Paraicoto | 9.550ºS; 77.791ºW | 11.47 | 3.6 |
| LSUMZ-86030 | *septentrionalis* | Ancash; 31 km (by road) E Paraicoto | 9.550ºS; 77.791ºW | 12.68 | 3.9 |
| LSUMZ-86031 | *septentrionalis* | Ancash; 31 km (by road) E Paraicoto | 9.550ºS; 77.791ºW | 12.40 | 3.6 |
| LSUMZ-86032 | *septentrionalis* | Ancash; 31 km (by road) E Paraicoto | 9.550ºS; 77.791ºW | 12.68 | 3.4 |
| LSUMZ-86033 | *septentrionalis* | Ancash; 31 km (by road) E Paraicoto | 9.550ºS; 77.791ºW | 12.85 | 3.7 |
| LSUMZ-86034 | *septentrionalis* | Ancash; 31 km (by road) E Paraicoto | 9.550ºS; 77.791ºW | 12.32 | 3.2 |
| LSUMZ-86035 | *septentrionalis* | Ancash; 31 km (by road) E Paraicoto | 9.550ºS; 77.791ºW | 12.12 | 3.1 |
| LSUMZ-86036 | *septentrionalis* | Ancash; 31 km (by road) E Paraicoto | 9.550ºS; 77.791ºW | 12.64 | 3.6 |
| LSUMZ-84491 | *septentrionalis* | Cajamarca; Cutervo | 6.390ºS; 78.803ºW | 12.20 | 3.9 |
| LSUMZ-84490 | *septentrionalis* | Cajamarca; Cutervo | 6.390ºS; 78.803ºW | 11.46 | 3.7 |
| LSUMZ-84492 | *septentrionalis* | Cajamarca; Cutervo | 6.390ºS; 78.803ºW | 11.5 | 4.2 |
| LSUMZ-84488 | *septentrionalis* | Cajamarca; 7 km N, 3 km E Chota | 6.498ºS; 78.624ºW | 11.34 | 3.5 |
| LSUMZ-84489 | *septentrionalis* | Cajamarca; 7 km N, 3 km E Chota | 6.498ºS; 78.624ºW | 11.89 | 3.9 |
| LSUMZ-91951 | *septentrionalis* | La Libertad; 22 road km W Shorey | 7.996ºS; 78.425ºW | 12.63 | 3.6 |
| LSUMZ-91952 | *septentrionalis* | La Libertad; Quebrada La Caldera, 7km NE Tayabamba | 8.228ºS; 77.260ºW | 12.13 | 3.6 |
| [MSB:BIRD:27001](http://arctos.database.museum/guid/MSB:Bird:27001)* | *septentrionalis* | Lima: 2.0 km E San Pedro de Casta | 11.758ºS; 76.584ºW |  |  |
| [MSB:BIRD:31016](http://arctos.database.museum/guid/MSB:Bird:31016)* | *septentrionalis* | Lima: 2.0 km E San Pedro de Casta | 11.758ºS; 76.584ºW |  |  |
| [MSB:BIRD:31038](http://arctos.database.museum/guid/MSB:Bird:31038)* | *septentrionalis* | Lima: 2.0 km E San Pedro de Casta | 11.758ºS; 76.584ºW |  |  |
| [MSB:BIRD:28467](http://arctos.database.museum/guid/MSB:Bird:28467)* | *septentrionalis* | Lima; dist. San Pedro de Casta; Pariangancha | 11.743ºS; 76.606ºW | 13.08 | 3.4 |
| FMNH-331184 | *smaragdinicollis* | Amazonas; 20km W Leymebamba | 6.781ºS; 77.915ºW | 10.49 | 3.8 |
| FMNH-331185 | *smaragdinicollis* | Amazonas; 20km W Leymebamba | 6.781ºS; 77.915ºW | 12.09 | 3.6 |
| FMNH-331187 | *smaragdinicollis* | Amazonas; 20km W Leymebamba | 6.781ºS; 77.915ºW | 10.99 | 4 |
| FMNH-331188 | *smaragdinicollis* | Amazonas; 20km W Leymebamba | 6.781ºS; 77.915ºW | 11.12 | 4 |
| LSUMZ-87552 | *smaragdinicollis* | Amazonas; Cordillera Colan, E La Peca | 5.572ºS; 78.278ºW | 9.67 | 3.0 |
| LSUMZ-87553 | *smaragdinicollis* | Amazonas; Cordillera Colan, E La Peca | 5.572ºS; 78.278ºW | 10.21 | 3.7 |
| LSUMZ-87554 | *smaragdinicollis* | Amazonas; Cordillera Colan, E La Peca | 5.572ºS; 78.278ºW | 10.50 | 3.4 |
| LSUMZ-87555 | *smaragdinicollis* | Amazonas; Cordillera Colan, E La Peca | 5.572ºS; 78.278ºW | 10.21 | 3.6 |
| LSUMZ-87558 | *smaragdinicollis* | Amazonas; Cordillera Colan, E La Peca | 5.572ºS; 78.278ºW | 11.91 | 3.5 |
| LSUMZ-87556 | *smaragdinicollis* | Amazonas; Cordillera Colan, E La Peca | 5.572ºS; 78.278ºW | 10.92 | 3.4 |
| LSUMZ-87557 | *smaragdinicollis* | Amazonas; Cordillera Colan, E La Peca | 5.572ºS; 78.278ºW | 10.71 | 3.8 |
| LSUMZ-87550 | *smaragdinicollis* | Amazonas; Cordillera Colan, E La Peca | 5.572ºS; 78.278ºW | 10.60 | 3.8 |
| LSUMZ-87551 | *smaragdinicollis* | Amazonas; Cordillera Colan, E La Peca | 5.572ºS; 78.278ºW | 10.42 | 3.4 |
| LSUMZ-80488 | *smaragdinicollis* | Amazonas; km404 on Balsas Leymebamba rd. | 6.764ºS; 77.861ºW | 11.96 | 3.5 |
| [MSB:BIRD:32672](http://arctos.database.museum/guid/MSB:Bird:32672)* | *smaragdinicollis* | Amazonas; Prov. Utcubamba, dist. Lonya Grande | 6.083ºS; 78.319ºW | 10.58 | 3.8 |
| [MSB:BIRD:33660](http://arctos.database.museum/guid/MSB:Bird:33660)* | *smaragdinicollis* | Apurímac; Puente Nununya | 14.679ºS; 73.148ºW |  |  |
| [MSB:BIRD:35855](http://arctos.database.museum/guid/MSB:Bird:35855)* | *smaragdinicollis* | Apurímac; Puente Nununya | 14.679ºS; 73.148ºW |  |  |
| [MSB:BIRD:33896](http://arctos.database.museum/guid/MSB:Bird:33896)* | *smaragdinicollis* | Apurímac; 5 km W Huanipaca, Ccocha | 13.487ºS; 72.982ºW | 13.32 | 3.35 |
| [MSB:BIRD:33910](http://arctos.database.museum/guid/MSB:Bird:33910)* | *smaragdinicollis* | Apurímac; 5 km W Huanipaca, Ccocha | 13.487ºS; 72.982ºW |  |  |
| [MSB:BIRD:33926](http://arctos.database.museum/guid/MSB:Bird:33926)* | *smaragdinicollis* | Apurímac; 5 km W Huanipaca, Ccocha | 13.487ºS; 72.982ºW | 13.68 | 3.5 |
| [MSB:BIRD:33933](http://arctos.database.museum/guid/MSB:Bird:33933)* | *smaragdinicollis* | Apurímac; 5 km W Huanipaca, Ccocha | 13.487ºS; 72.982ºW |  |  |
| [MSB:BIRD:33936](http://arctos.database.museum/guid/MSB:Bird:33936)* | *smaragdinicollis* | Apurímac; 5 km W Huanipaca, Ccocha | 13.487ºS; 72.982ºW |  |  |
| [MSB:BIRD:33944](http://arctos.database.museum/guid/MSB:Bird:33944)* | *smaragdinicollis* | Apurímac; 5 km W Huanipaca, Ccocha | 13.487ºS; 72.982ºW | 12.49 | 4.07 |
| [MSB:BIRD:33954](http://arctos.database.museum/guid/MSB:Bird:33954)* | *smaragdinicollis* | Apurímac; 5 km W Huanipaca, Ccocha | 13.487ºS; 72.982ºW | 13.26 | 3.87 |
| [MSB:BIRD:33957](http://arctos.database.museum/guid/MSB:Bird:33957)* | *smaragdinicollis* | Apurímac; 5 km W Huanipaca, Ccocha | 13.487ºS; 72.982ºW |  |  |
| [MSB:BIRD:33964](http://arctos.database.museum/guid/MSB:Bird:33964)* | *smaragdinicollis* | Apurímac; 5 km W Huanipaca, Ccocha | 13.487ºS; 72.982ºW | 12.49 | 3.65 |
| [MSB:BIRD:33986](http://arctos.database.museum/guid/MSB:Bird:33986)* | *smaragdinicollis* | Apurímac; 5 km W Huanipaca, Ccocha | 13.487ºS; 72.982ºW |  |  |
| [MSB:BIRD:33987](http://arctos.database.museum/guid/MSB:Bird:33987)* | *smaragdinicollis* | Apurímac; 5 km W Huanipaca, Ccocha | 13.487ºS; 72.982ºW | 13.54 | 3.91 |
| [MSB:BIRD:33990](http://arctos.database.museum/guid/MSB:Bird:33990)* | *smaragdinicollis* | Apurímac; 5 km W Huanipaca, Ccocha | 13.487ºS; 72.982ºW | 12.79 | 4.0 |
| [MSB:BIRD:33994](http://arctos.database.museum/guid/MSB:Bird:33994)* | *smaragdinicollis* | Apurímac; 5 km W Huanipaca, Ccocha | 13.487ºS; 72.982ºW |  |  |
| [MSB:BIRD:34007](http://arctos.database.museum/guid/MSB:Bird:34007)* | *smaragdinicollis* | Apurímac; 5 km W Huanipaca, Ccocha | 13.487ºS; 72.982ºW | 13.62 | 3.64 |
| [MSB:BIRD:34008](http://arctos.database.museum/guid/MSB:Bird:34008)* | *smaragdinicollis* | Apurímac; 5 km W Huanipaca, Ccocha | 13.487ºS; 72.982ºW |  |  |
| [MSB:BIRD:34015](http://arctos.database.museum/guid/MSB:Bird:34015)* | *smaragdinicollis* | Apurímac; 5 km W Huanipaca, Ccocha | 13.487ºS; 72.982ºW |  |  |
| [MSB:BIRD:34016](http://arctos.database.museum/guid/MSB:Bird:34016)* | *smaragdinicollis* | Apurímac; 5 km W Huanipaca, Ccocha | 13.487ºS; 72.982ºW | 12.82 | 3.84 |
| [MSB:BIRD:34022](http://arctos.database.museum/guid/MSB:Bird:34022)* | *smaragdinicollis* | Apurímac; 5 km W Huanipaca, Ccocha | 13.487ºS; 72.982ºW | 13.06 | 3.49 |
| [MSB:BIRD:33897](http://arctos.database.museum/guid/MSB:Bird:33897) | *smaragdinicollis* | Apurímac; 5 km W Huanipaca, Ccocha | 13.487ºS; 72.982ºW | 13.02 | 3.43 |
| [MSB:BIRD:33911](http://arctos.database.museum/guid/MSB:Bird:33911) | *smaragdinicollis* | Apurímac; 5 km W Huanipaca, Ccocha | 13.487ºS; 72.982ºW | 13.63 | 4.10 |
| CORBIDI-16999* | *smaragdinicollis* | Ayacucho; 2 km S Ccano | 12.785ºS; 73.995ºW | 11.92 | 3.75 |
| CORBIDI-17168* | *smaragdinicollis* | Ayacucho; 2 km S Ccano | 12.785ºS; 73.995ºW | 11.04 | 3.6 |
| KUMNH-25113* | *smaragdinicollis* | Ayacucho; 2 km S Ccano | 12.785ºS; 73.995ºW |  |  |
| KUMNH-25141* | *smaragdinicollis* | Ayacucho; 2 km S Ccano | 12.785ºS; 73.995ºW |  |  |
| FMNH-433153** | *smaragdinicollis* | Cusco, Paucartambo, La Esperanza | 13.167ºS; 71.600ºW | 11.41 | 3.4 |
| FMNH-433154** | *smaragdinicollis* | Cusco, Paucartambo, La Esperanza | 13.167ºS; 71.600ºW | 11.13 | 3.5 |
| FMNH-433156** | *smaragdinicollis* | Cusco, Paucartambo, La Esperanza | 13.167ºS; 71.600ºW | 11.1 | 3.4 |
| FMNH-433157** | *smaragdinicollis* | Cusco, Paucartambo, La Esperanza | 13.167ºS; 71.600ºW | 11.63 | 3.7 |
| FMNH-433158** | *smaragdinicollis* | Cusco, Paucartambo, La Esperanza | 13.167ºS; 71.600ºW |  |  |
| FMNH-433160 | *smaragdinicollis* | Cusco, Paucartambo, La Esperanza | 13.167ºS; 71.600ºW | 12.69 | 3.5 |
| FMNH-429932** | *smaragdinicollis* | Cusco; Paucartambo, Pillahuata | 13.162ºS; 71.594ºW | 11.36 | 3.63 |
| FMNH-429933** | *smaragdinicollis* | Cusco, Paucartambo, Pillahuata | 13.162ºS; 71.594ºW | 12.18 | 3.0 |
| FMNH-429934** | *smaragdinicollis* | Cusco; Paucartambo, Pillahuata | 13.162ºS; 71.594ºW |  |  |
| FMNH-429935 | *smaragdinicollis* | Cusco, Paucartambo, Pillahuata | 13.162ºS; 71.594ºW | 11.85 | 3.0 |
| FMNH-397844 | *smaragdinicollis* | Cusco, Paucartambo, Puesto de Vigilancia de Acjanaco | 13.200ºS; 71.618ºW | 12.12 | 3.5 |
| FMNH-397845 | *smaragdinicollis* | Cusco, Paucartambo, Puesto de Vigilancia de Acjanaco | 13.200ºS; 71.618ºW | 11.63 | 3.75 |
| FMNH-397849* | *smaragdinicollis* | Cusco, Paucartambo, Puesto de Vigilancia de Acjanaco | 13.200ºS; 71.618ºW |  |  |
| FMNH-397850 | *smaragdinicollis* | Cusco, Paucartambo, Puesto de Vigilancia de Acjanaco | 13.200ºS; 71.618ºW | 11.62 | 3.75 |
| FMNH-397851* | *smaragdinicollis* | Cusco, Paucartambo, Puesto de Vigilancia de Acjanaco | 13.200ºS; 71.618ºW |  |  |
| FMNH-397852 | *smaragdinicollis* | Cusco, Paucartambo, Puesto de Vigilancia de Acjanaco | 13.200ºS; 71.618ºW | 11.59 | 3.5 |
| FMNH-291606 | *smaragdinicollis* | Cusco, Pillahuata, km#126 on Cosñipata Hwy. | 13.162ºS; 71.594ºW | 11.10 | 3.6 |
| FMNH-311776 | *smaragdinicollis* | Cusco, Pillahuata, km#126 on Cosñipata Hwy. | 13.162ºS; 71.594ºW | 11.46 | 3.5 |
| FMNH-311778 | *smaragdinicollis* | Cusco, Pillahuata, km#126 on Cosñipata Hwy. | 13.162ºS; 71.594ºW | 11.57 | 3.6 |
| [MSB:BIRD:33567](http://arctos.database.museum/guid/MSB:Bird:33567) | *smaragdinicollis* | Cusco, Prov. Marcapata, Distrito Corani, Chile Chile | 13.547ºS; 70.892ºW | 12.54 | 3.68 |
| [MSB:BIRD:35827](http://arctos.database.museum/guid/MSB:Bird:35827)** | *smaragdinicollis* | Cusco, Prov. Paucartambo; 9.6km NE Paucartambo | 13.221ºS; 71.628ºW | 12.67 | 3.79 |
| [MSB:BIRD:35829](http://arctos.database.museum/guid/MSB:Bird:35829)** | *smaragdinicollis* | Cusco, Prov. Paucartambo; 9.6km NE Paucartambo | 13.221ºS; 71.628ºW | 12.3 | 3.58 |
| [MSB:BIRD:35830](http://arctos.database.museum/guid/MSB:Bird:35830)** | *smaragdinicollis* | Cusco, Prov. Paucartambo; 9.6km NE Paucartambo | 13.221ºS; 71.628ºW | 12.63 | 3.52 |
| [MSB:BIRD:35820](http://arctos.database.museum/guid/MSB:Bird:35820)** | *smaragdinicollis* | Cusco; 2.5km E Carrizales, Puente de la Sirena | 13.085ºS; 72.367ºW | 11.97 | 3.89 |
| [MSB:BIRD:35803](http://arctos.database.museum/guid/MSB:Bird:35803)** | *smaragdinicollis* | Cusco; 3.7km SE Carrizales | 13.107ºS; 72.357ºW | 11.84 | 3.52 |
| [MSB:BIRD:35804](http://arctos.database.museum/guid/MSB:Bird:35804)** | *smaragdinicollis* | Cusco; 3.7km SE Carrizales | 13.107ºS; 72.357ºW | 12.08 | 3.64 |
| [MSB:BIRD:35807](http://arctos.database.museum/guid/MSB:Bird:35807)** | *smaragdinicollis* | Cusco; 3.7km SE Carrizales | 13.107ºS; 72.357ºW | 11.4 | 3.6 |
| [MSB:BIRD:35808](http://arctos.database.museum/guid/MSB:Bird:35808)** | *smaragdinicollis* | Cusco; 3.7km SE Carrizales | 13.107ºS; 72.357ºW | 11.28 | 3.86 |
| [MSB:BIRD:36590](http://arctos.database.museum/guid/MSB:Bird:36590)** | *smaragdinicollis* | Cusco; Prov. Paucartambo, Distrito Challabamba | 13.203ºS; 71.627ºW | 12.01 | 3.62 |
| [MSB:BIRD:36593](http://arctos.database.museum/guid/MSB:Bird:36593)** | *smaragdinicollis* | Cusco; Prov. Paucartambo, Distrito Challabamba | 13.203ºS; 71.627ºW | 11.5 | 3.7 |
| [MSB:BIRD:36594](http://arctos.database.museum/guid/MSB:Bird:36594)** | *smaragdinicollis* | Cusco; Prov. Paucartambo, Distrito Challabamba | 13.203ºS; 71.627ºW | 12.3 | 3.82 |
| [MSB:BIRD:36595](http://arctos.database.museum/guid/MSB:Bird:36595)** | *smaragdinicollis* | Cusco; Prov. Paucartambo, Distrito Challabamba | 13.203ºS; 71.627ºW | 12.24 | 3.33 |
| [MSB:BIRD:35674](http://arctos.database.museum/guid/MSB:Bird:35674)** | *smaragdinicollis* | Cusco; Prov. Paucartambo, distrito Pillahuata | 13.162ºS; 71.594ºW | 11.94 | 3.36 |
| [MSB:BIRD:35676](http://arctos.database.museum/guid/MSB:Bird:35676)** | *smaragdinicollis* | Cusco; Prov. Paucartambo, distrito Pillahuata | 13.162ºS; 71.594ºW | 12.76 | 3.39 |
| [MSB:BIRD:35678](http://arctos.database.museum/guid/MSB:Bird:35678)** | *smaragdinicollis* | Cusco; Prov. Paucartambo, distrito Pillahuata | 13.162ºS; 71.594ºW | 12.87 | 3.49 |
| [MSB:BIRD:35679](http://arctos.database.museum/guid/MSB:Bird:35679)** | *smaragdinicollis* | Cusco; Prov. Paucartambo, distrito Pillahuata | 13.162ºS; 71.594ºW | 12.88 | 3.47 |
| [MSB:BIRD:35683](http://arctos.database.museum/guid/MSB:Bird:35683)** | *smaragdinicollis* | Cusco; Prov. Paucartambo, distrito Pillahuata | 13.162ºS; 71.594ºW | 11.43 | 3.63 |
| [MSB:BIRD:27214](http://arctos.database.museum/guid/MSB:Bird:27214)** | *smaragdinicollis* | Cusco; Prov. Urubamba | 13.267ºS; 72.182ºW | 13.7 | 3.6 |
| [MSB:BIRD:33073](http://arctos.database.museum/guid/MSB:Bird:33073)** | *smaragdinicollis* | Cusco; Prov. Urubamba | 13.267ºS; 72.182ºW | 12.88 | 3.35 |
| [MSB:BIRD:36596](http://arctos.database.museum/guid/MSB:Bird:36596)** | *smaragdinicollis* | Cusco; Prov. Urubamba, near Carrizales | 13.114ºS; 72.345ºW | 12.03 | 3.6 |
| [MSB:BIRD:27185](http://arctos.database.museum/guid/MSB:Bird:27185)** | *smaragdinicollis* | Cusco; Prov. Urubamba, 7.9 km NW Urubamba | 13.250ºS; 72.169ºW | 13.25 | 3.8 |
| [MSB:BIRD:27186](http://arctos.database.museum/guid/MSB:Bird:27186)** | *smaragdinicollis* | Cusco; Prov. Urubamba, 7.9 km NW Urubamba | 13.250ºS; 72.169ºW | 13.18 | 3.8 |
| [MSB:BIRD:27205](http://arctos.database.museum/guid/MSB:Bird:27205)** | *smaragdinicollis* | Cusco; Prov. Urubamba, 7.9 km NW Urubamba | 13.250ºS; 72.169ºW | 14.31 | 3.8 |
| [MSB:BIRD:27216](http://arctos.database.museum/guid/MSB:Bird:27216)** | *smaragdinicollis* | Cusco; Prov. Urubamba, 7.9 km NW Urubamba | 13.250ºS; 72.169ºW | 12.7 | 3.7 |
| [MSB:BIRD:31191](http://arctos.database.museum/guid/MSB:Bird:31191)** | *smaragdinicollis* | Cusco; Prov. Urubamba, 7.9 km NW Urubamba | 13.250ºS; 72.169ºW | 14.39 | 3.7 |
| [MSB:BIRD:31192](http://arctos.database.museum/guid/MSB:Bird:31192)** | *smaragdinicollis* | Cusco; Prov. Urubamba, 7.9 km NW Urubamba | 13.250ºS; 72.169ºW | 13.36 | 3.4 |
| [MSB:BIRD:31193](http://arctos.database.museum/guid/MSB:Bird:31193)** | *smaragdinicollis* | Cusco; Prov. Urubamba, 7.9 km NW Urubamba | 13.250ºS; 72.169ºW | 13.89 | 3.6 |
| [MSB:BIRD:31195](http://arctos.database.museum/guid/MSB:Bird:31195)** | *smaragdinicollis* | Cusco; Prov. Urubamba, 7.9 km NW Urubamba | 13.250ºS; 72.169ºW | 13.00 | 3.3 |
| [MSB:BIRD:31196](http://arctos.database.museum/guid/MSB:Bird:31196)** | *smaragdinicollis* | Cusco; Prov. Urubamba, 7.9 km NW Urubamba | 13.250ºS; 72.169ºW | 14.86 | 3.3 |
| [MSB:BIRD:31197](http://arctos.database.museum/guid/MSB:Bird:31197)** | *smaragdinicollis* | Cusco; Prov. Urubamba, 7.9 km NW Urubamba | 13.250ºS; 72.169ºW | 14.14 | 3.8 |
| [MSB:BIRD:31198](http://arctos.database.museum/guid/MSB:Bird:31198)** | *smaragdinicollis* | Cusco; Prov. Urubamba, 7.9 km NW Urubamba | 13.250ºS; 72.169ºW | 14.03 | 3.6 |
| [MSB:BIRD:31199](http://arctos.database.museum/guid/MSB:Bird:31199)** | *smaragdinicollis* | Cusco; Prov. Urubamba, 7.9 km NW Urubamba | 13.250ºS; 72.169ºW | 13.84 | 3.5 |
| [MSB:BIRD:31200](http://arctos.database.museum/guid/MSB:Bird:31200)** | *smaragdinicollis* | Cusco; Prov. Urubamba, 7.9 km NW Urubamba | 13.250ºS; 72.169ºW | 14.30 | 3.8 |
| [MSB:BIRD:31201](http://arctos.database.museum/guid/MSB:Bird:31201)** | *smaragdinicollis* | Cusco; Prov. Urubamba, 7.9 km NW Urubamba | 13.250ºS; 72.169ºW | 14.33 | 3.6 |
| [MSB:BIRD:31202](http://arctos.database.museum/guid/MSB:Bird:31202)** | *smaragdinicollis* | Cusco; Prov. Urubamba, 7.9 km NW Urubamba | 13.250ºS; 72.169ºW | 13.15 | 3.6 |
| [MSB:BIRD:31203](http://arctos.database.museum/guid/MSB:Bird:31203)** | *smaragdinicollis* | Cusco; Prov. Urubamba, 7.9 km NW Urubamba | 13.250ºS; 72.169ºW | 14.62 | 3.5 |
| [MSB:BIRD:33069](http://arctos.database.museum/guid/MSB:Bird:33069)** | *smaragdinicollis* | Cusco; Prov. Urubamba, Ollantaytambo | 13.194ºS; 72.233ºW | 13.17 | 3.0 |
| [MSB:BIRD:27219](http://arctos.database.museum/guid/MSB:Bird:27219)** | *smaragdinicollis* | Cusco; Prov. Urubamba; 7.9km NW Urubamba | 13.250ºS; 72.169ºW | 14.34 | 3.5 |
| [MSB:BIRD:27225](http://arctos.database.museum/guid/MSB:Bird:27225)** | *smaragdinicollis* | Cusco; Prov. Urubamba; 7.9km NW Urubamba | 13.250ºS; 72.169ºW | 13.45 | 3.2 |
| LSUMZ-73903 | *smaragdinicollis* | Huanuco; base of Bosque Tapra above Acomayo | 9.717ºS; 76.065ºW | 10.72 | 3 |
| [MSB:BIRD:31567](http://arctos.database.museum/guid/MSB:Bird:31567)* | *smaragdinicollis* | Huanuco; Prov. Pachitea, Chincho Ocomayo, Carpish Tunnel | 9.727ºS; 76.106ºW | 10.89 | 3.55 |
| [MSB:BIRD:31589](http://arctos.database.museum/guid/MSB:Bird:31589)* | *smaragdinicollis* | Huanuco; Prov. Pachitea, Chincho Ocomayo, Carpish Tunnel | 9.727ºS; 76.106ºW | 10.73 | 3.81 |
| [MSB:BIRD:31631](http://arctos.database.museum/guid/MSB:Bird:31631)* | *smaragdinicollis* | Huanuco; Prov. Pachitea, Chincho Ocomayo, Carpish Tunnel | 9.727ºS; 76.106ºW | 10.92 | 4.3 |
| [MSB:BIRD:31649](http://arctos.database.museum/guid/MSB:Bird:31649)* | *smaragdinicollis* | Huanuco; Prov. Pachitea, Chincho Ocomayo, Carpish Tunnel | 9.727ºS; 76.106ºW | 10.82 | 3.5 |
| [MSB:BIRD:31556](http://arctos.database.museum/guid/MSB:Bird:31556) | *smaragdinicollis* | Huanuco; Prov. Pachitea, Chincho Ocomayo, Carpish Tunnel | 9.727ºS; 76.106ºW | 10.16 | 3.99 |
| [MSB:BIRD:31633](http://arctos.database.museum/guid/MSB:Bird:31633) | *smaragdinicollis* | Huanuco; Prov. Pachitea, Chincho Ocomayo, Carpish Tunnel | 9.727ºS; 76.106ºW | 11.33 | 3.76 |
| [MSB:BIRD:31555](http://arctos.database.museum/guid/MSB:Bird:31555)* | *smaragdinicollis* | Huanuco; Prov. Pachitea; Chincho ocomayo, Carpish Tunnel | 9.727ºS; 76.106ºW | 10.92 | 3.71 |
| [MSB:BIRD:31164](http://arctos.database.museum/guid/MSB:Bird:31164)* | *smaragdinicollis* | Junin; | 11.489ºS; 74.896ºW |  |  |
| [MSB:BIRD:31169](http://arctos.database.museum/guid/MSB:Bird:31169)* | *smaragdinicollis* | Junin; | 11.489ºS; 74.896ºW |  |  |
| [MSB:BIRD:31179](http://arctos.database.museum/guid/MSB:Bird:31179)* | *smaragdinicollis* | Junin; | 11.489ºS; 74.896ºW |  |  |
| LSUMZ-127598 | *smaragdinicollis* | Junin; Pampa Huasi, SE Calabaza | 11.535ºS; 74.732ºW | 11.78 | 4 |
| LSUMZ-127599 | *smaragdinicollis* | Junin; Pampa Huasi, SE Calabaza | 11.535ºS; 74.732ºW | 11.01 | 3.2 |
| LSUMZ-127588 | *smaragdinicollis* | Junin; via Satipo, Chanchuleo, SE de Calabaza | 11.535ºS; 74.732ºW | 10.83 | 3.2 |
| LSUMZ-127590 | *smaragdinicollis* | Junin; via Satipo, Chanchuleo, SE de Calabaza | 11.535ºS; 74.732ºW | 10.74 | 3.2 |
| LSUMZ-127592 | *smaragdinicollis* | Junin; via Satipo, Chanchuleo, SE de Calabaza | 11.535ºS; 74.732ºW | 10.56 | 3 |
| LSUMZ-127594 | *smaragdinicollis* | Junin; via Satipo, Chanchuleo, SE de Calabaza | 11.535ºS; 74.732ºW | 10.72 | 4 |
| LSUMZ-127595 | *smaragdinicollis* | Junin; via Satipo, Chanchuleo, SE de Calabaza | 11.535ºS; 74.732ºW | 11.37 | 3 |
| LSUMZ-127596 | *smaragdinicollis* | Junin; via Satipo, Chanchuleo, SE de Calabaza | 11.535ºS; 74.732ºW | 10.27 | 3.6 |
| LSUMZ-91954 | *smaragdinicollis* | La Libertad; Mashua, E Tayabamba on trail to Ongon | 8.200ºS; 77.230ºW | 11.19 | 4.4 |
| [MSB:BIRD:35644](http://arctos.database.museum/guid/MSB:Bird:35644)* | *smaragdinicollis* | Pasco; Huancabamba, Yanachaga | 10.390ºS; 75.470ºW |  |  |
| LSUMZ-128363 | *smaragdinicollis* | Pasco; 2 km NW Punta de Saria on Pozuzo-Chaglla trail | 9.900ºS; 75.730ºW | 12.24 | 4.8 |
| LSUMZ-128364 | *smaragdinicollis* | Pasco; 2 km NW Punta de Saria on Pozuzo-Chaglla trail | 9.900ºS; 75.730ºW | 10.35 | 3.8 |
| LSUMZ-105801 | *smaragdinicollis* | Pasco; Cumbre de Ollon, ca 12 km E Oxapampa | 10.577ºS: 75.296ºW | 10.93 | 3.6 |
| LSUMZ-128352 | *smaragdinicollis* | Pasco; Millpo, E Tambo de Vacas on Pozuzo-Chaglla trail | 9.900ºS; 75.730ºW | 11.08 | 3.7 |
| LSUMZ-128353 | *smaragdinicollis* | Pasco; Millpo, E Tambo de Vacas on Pozuzo-Chaglla trail | 9.900ºS; 75.730ºW | 11.64 | 4 |
| LSUMZ-128354 | *smaragdinicollis* | Pasco; Millpo, E Tambo de Vacas on Pozuzo-Chaglla trail | 9.900ºS; 75.730ºW | 11.03 | 3.8 |
| LSUMZ-128355 | *smaragdinicollis* | Pasco; Millpo, E Tambo de Vacas on Pozuzo-Chaglla trail | 9.900ºS; 75.730ºW | 10.9 | 3.9 |
| LSUMZ-128356 | *smaragdinicollis* | Pasco; Millpo, E Tambo de Vacas on Pozuzo-Chaglla trail | 9.900ºS; 75.730ºW | 10.86 | 3.3 |
| LSUMZ-128357 | *smaragdinicollis* | Pasco; Millpo, E Tambo de Vacas on Pozuzo-Chaglla trail | 9.900ºS; 75.730ºW | 11.01 | 3.7 |
| LSUMZ-128358 | *smaragdinicollis* | Pasco; Millpo, E Tambo de Vacas on Pozuzo-Chaglla trail | 9.900ºS; 75.730ºW | 10.77 | 3.7 |
| LSUMZ-128359 | *smaragdinicollis* | Pasco; Millpo, E Tambo de Vacas on Pozuzo-Chaglla trail | 9.900ºS; 75.730ºW | 9.48 | 3.7 |
| LSUMZ-128360 | *smaragdinicollis* | Pasco; Millpo, E Tambo de Vacas on Pozuzo-Chaglla trail | 9.900ºS; 75.730ºW | 10.23 | 3.5 |
| LSUMZ-128361 | *smaragdinicollis* | Pasco; Millpo, E Tambo de Vacas on Pozuzo-Chaglla trail | 9.900ºS; 75.730ºW | 11.02 | 3.5 |
| LSUMZ-128362 | *smaragdinicollis* | Pasco; Millpo, E Tambo de Vacas on Pozuzo-Chaglla trail | 9.900ºS; 75.730ºW | 11.13 | 3.4 |
| KUMNH-21169* | *smaragdinicollis* | Puno; Sina | 14.490ºS; 69.280ºW |  |  |
| KUMNH-21195* | *smaragdinicollis* | Puno; Sina | 14.490ºS; 69.280ºW |  |  |
| LSUMZ-98177 | *smaragdinicollis* | Puno; Valcon, 5 km NNW Quiaca | 14.458ºS; 69.420ºW | 10.91 | 3 |
| LSUMZ-98180 | *smaragdinicollis* | Puno; Valcon, 5 km NNW Quiaca | 14.458ºS; 69.420ºW | 10.87 | 3.2 |
| LSUMZ-98181 | *smaragdinicollis* | Puno; Valcon, 5 km NNW Quiaca | 14.458ºS; 69.420ºW | 10.77 | 3.4 |
| LSUMZ-98182 | *smaragdinicollis* | Puno; Valcon, 5 km NNW Quiaca | 14.458ºS; 69.420ºW | 11.66 | 3.2 |
| LSUMZ-98183 | *smaragdinicollis* | Puno; Valcon, 5 km NNW Quiaca | 14.458ºS; 69.420ºW | 11.27 | 3.4 |
| LSUMZ-98184 | *smaragdinicollis* | Puno; Valcon, 5 km NNW Quiaca | 14.458ºS; 69.420ºW | 11.69 | 3.4 |
| LSUMZ-98185 | *smaragdinicollis* | Puno; Valcon, 5 km NNW Quiaca | 14.458ºS; 69.420ºW | 11.36 | 4 |
| LSUMZ-173884 | *smaragdinicollis* | San Martin; ca. km ENE Florida | 5.723ºS; 77.750ºW | 10.14 | 3.6 |
| LSUMZ-173885* | *smaragdinicollis* | San Martin; ca. km ENE Florida | 5.723ºS; 77.750ºW | 10.46 | 3.1 |
| LSUMZ-B43683* | *smaragdinicollis* | San Martin; ca. km ENE Florida | 5.723ºS; 77.750ºW |  |  |
| LSUMZ-104463 | *smaragdinicollis* | San Martin; Puerta del Monte, ca. 30km NE Los Alisos | 7.529ºS; 77.480ºW | 9.62 | 3.3 |
| CORBIDI-AQ 222 | *tyrianthina* | Cajamarca; prov. Chota; distr. Queracoto, La Granja, Pagaibamba | 6.405ºS; 79.082ºW | 10.33 | 3.1 |
| CORBIDI-AQ 227 | *tyrianthina* | Cajamarca; prov. Chota; distr. Queracoto, La Granja, Pagaibamba | 6.405ºS; 79.082ºW | 10.68 | 3.1 |
| CORBIDI-AQ 228 | *tyrianthina* | Cajamarca; prov. Chota; distr. Queracoto, La Granja, Pagaibamba | 6.405ºS; 79.082ºW | 10.04 | 3.9 |
| CORBIDI-AQ 231 | *tyrianthina* | Cajamarca; prov. Chota; distr. Queracoto, La Granja, Pagaibamba | 6.405ºS; 79.082ºW | 8.89 | 3.3 |
| CORBIDI-AQ 234 | *tyrianthina* | Cajamarca; prov. Chota; distr. Queracoto, La Granja, Pagaibamba | 6.405ºS; 79.082ºW | 11.08 | 3.1 |
| CORBIDI-AQ 245 | *tyrianthina* | Cajamarca; prov. Chota; distr. Queracoto, La Granja, Pagaibamba | 6.405ºS; 79.082ºW | 12.18 | 3.3 |
| CORBIDI-AQ 263 | *tyrianthina* | Cajamarca; prov. Chota; distr. Queracoto, La Granja, Pagaibamba | 6.405ºS; 79.082ºW | 10.45 | 3.2 |
| CORBIDI-AQ 265 | *tyrianthina* | Cajamarca; prov. Chota; distr. Queracoto, La Granja, Pagaibamba | 6.405ºS; 79.082ºW | 10.62 | 3.9 |
| CORBIDI-AQ 266 | *tyrianthina* | Cajamarca; prov. Chota; distr. Queracoto, La Granja, Pagaibamba | 6.405ºS; 79.082ºW | 10.39 | 2.9 |
| CORBIDI-AQ 276 | *tyrianthina* | Cajamarca; prov. Chota; distr. Queracoto, La Granja, Pagaibamba | 6.405ºS; 79.082ºW | 10.47 | 3.5 |
| CORBIDI-JAN 808 | *tyrianthina* | Cajamarca; prov. Chota; distr. Queracoto, La Granja, Pagaibamba | 6.405ºS; 79.082ºW | 10.98 | 3 |
| CORBIDI-JAN 809 | *tyrianthina* | Cajamarca; prov. Chota; distr. Queracoto, La Granja, Pagaibamba | 6.405ºS; 79.082ºW | 11.36 | 3.3 |
| CORBIDI-JAN 810 | *tyrianthina* | Cajamarca; prov. Chota; distr. Queracoto, La Granja, Pagaibamba | 6.405ºS; 79.082ºW | 11 | 3.6 |
| CORBIDI-JAN 811 | *tyrianthina* | Cajamarca; prov. Chota; distr. Queracoto, La Granja, Pagaibamba | 6.405ºS; 79.082ºW | 11.03 | 3.5 |
| CORBIDI-JNZ 335 | *tyrianthina* | Cajamarca; prov. Chota; distr. Queracoto, La Granja, Pagaibamba | 6.405ºS; 79.082ºW | 11.99 | 3.2 |
| CORBIDI-JNZ 348 | *tyrianthina* | Cajamarca; prov. Chota; distr. Queracoto, La Granja, Pagaibamba | 6.405ºS; 79.082ºW | 10.52 | 3.2 |
| CORBIDI-JNZ 357 | *tyrianthina* | Cajamarca; prov. Chota; distr. Queracoto, La Granja, Pagaibamba | 6.405ºS; 79.082ºW | 11.16 | 3 |
| CORBIDI-LA 007 | *tyrianthina* | Cajamarca; prov. Chota; distr. Queracoto, La Granja, Pagaibamba | 6.405ºS; 79.082ºW | 11.04 | 3.7 |
| CORBIDI-LA 010 | *tyrianthina* | Cajamarca; prov. Chota; distr. Queracoto, La Granja, Pagaibamba | 6.405ºS; 79.082ºW | 11.41 | 3 |
| CORBIDI-LA 008 | *tyrianthina* | Cajamarca; prov. Chota; distr. Queracoto, La Granja, Pagaibamba | 6.405ºS; 79.082ºW | 11.44 | 2.9 |
| LSUMZ-178963 | *tyrianthina* | Cajamarca; Hito Jesus | 4.894ºS; 78.915ºW | 10.68 | 3.4 |
| LSUMZ-178964 | *tyrianthina* | Cajamarca; Hito Jesus | 4.894ºS; 78.915ºW | 9.73 | 3.6 |
| LSUMZ-169715 | *tyrianthina* | Cajamarca; Quebrada Lanchal, ca. 8km ESE Sallique | 5.690ºS; 79.250ºW | 10.22 | 3.4 |
| LSUMZ-169716 | *tyrianthina* | Cajamarca; Quebrada Lanchal, ca. 8km ESE Sallique | 5.690ºS; 79.250ºW | 9.98 | 3.2 |
| LSUMZ-169717 | *tyrianthina* | Cajamarca; Quebrada Lanchal, ca. 8km ESE Sallique | 5.690ºS; 79.250ºW | 10.84 | 3 |
| LSUMZ-169718 | *tyrianthina* | Cajamarca; Quebrada Lanchal, ca. 8km ESE Sallique | 5.690ºS; 79.250ºW | 11.33 | 3.1 |
| LSUMZ-169719 | *tyrianthina* | Cajamarca; Quebrada Lanchal, ca. 8km ESE Sallique | 5.690ºS; 79.250ºW | 11.23 | 3 |
| LSUMZ-169720 | *tyrianthina* | Cajamarca; Quebrada Lanchal, ca. 8km ESE Sallique | 5.690ºS; 79.250ºW | 10.4 | 3.5 |
| LSUMZ-169721* | *tyrianthina* | Cajamarca; Quebrada Lanchal, ca. 8km ESE Sallique | 5.690ºS; 79.250ºW | 10.82 | 3.5 |
| LSUMZ-169722* | *tyrianthina* | Cajamarca; Quebrada Lanchal, ca. 8km ESE Sallique | 5.690ºS; 79.250ºW | 10.24 | 3.2 |
| LSUMZ-169723 | *tyrianthina* | Cajamarca; Quebrada Lanchal, ca. 8km ESE Sallique | 5.690ºS; 79.250ºW | 10.12 | 3.4 |
| LSUMZ-169724* | *tyrianthina* | Cajamarca; Quebrada Lanchal, ca. 8km ESE Sallique | 5.690ºS; 79.250ºW | 10.44 | 3.5 |
| LSUMZ-B32073* | *tyrianthina* | Cajamarca; Quebrada Lanchal, ca. 8km ESE Sallique | 5.690ºS; 79.250ºW | 9.91 | 3.9 |
| LSUMZ-B32431* | *tyrianthina* | Cajamarca; Quebrada Lanchal, ca. 8km ESE Sallique | 5.690ºS; 79.250ºW | 10.38 | 3.4 |
| LSUMZ-B31842 | *tyrianthina* | Cajamarca; Quebrada Lanchal, ca. 8km ESE Sallique | 5.690ºS; 79.250ºW | 10.44 | 3.6 |
| LSUMZ-B31788 | *tyrianthina* | Cajamarca; Quebrada Lanchal, ca. 8km ESE Sallique | 5.690ºS; 79.250ºW | 11.3 | 3.2 |
| LSUMZ-B32100 | *tyrianthina* | Cajamarca; Quebrada Lanchal, ca. 8km ESE Sallique | 5.690ºS; 79.250ºW | 10.82 | 3.4 |
| LSUMZ-B31974* | *tyrianthina* | Cajamarca; Quebrada Lanchal, ca. 8km ESE Sallique | 5.690ºS; 79.250ºW |  |  |
| LSUMZ-B 32101* | *tyrianthina* | Cajamarca; Quebrada Lanchal, ca. 8km ESE Sallique | 5.690ºS; 79.250ºW |  |  |
| LSUMZ-B 32520* | *tyrianthina* | Cajamarca; Quebrada Lanchal, ca. 8km ESE Sallique | 5.690ºS; 79.250ºW |  |  |
| [MSB:BIRD:28081](http://arctos.database.museum/guid/MSB:Bird:28081)* | *tyrianthina* | Lambayeque; | 6.237ºS; 79.234ºW | 10.69 | 3.7 |
| [MSB:BIRD:28083](http://arctos.database.museum/guid/MSB:Bird:28083)* | *tyrianthina* | Lambayeque; | 6.237ºS; 79.234ºW | 11.29 | 4.05 |
| [MSB:BIRD:28084](http://arctos.database.museum/guid/MSB:Bird:28084)* | *tyrianthina* | Lambayeque; | 6.237ºS; 79.234ºW | 11.28 | 4.07 |
| [MSB:BIRD:28124](http://arctos.database.museum/guid/MSB:Bird:28124)* | *tyrianthina* | Lambayeque; | 6.237ºS; 79.234ºW | 11.2 | 3.9 |
| [MSB:BIRD:31339](http://arctos.database.museum/guid/MSB:Bird:31339)* | *tyrianthina* | Lambayeque; | 6.237ºS; 79.234ºW |  |  |
| [MSB:BIRD:28203](http://arctos.database.museum/guid/MSB:Bird:28203) | *tyrianthina* | Lambayeque; | 6.237ºS; 79.234ºW | 11.82 | 3.56 |
| [MSB:BIRD:28207](http://arctos.database.museum/guid/MSB:Bird:28207) | *tyrianthina* | Lambayeque; | 6.237ºS; 79.234ºW | 11.99 | 3.78 |
| [MSB:BIRD:28209](http://arctos.database.museum/guid/MSB:Bird:28209) | *tyrianthina* | Lambayeque; | 6.237ºS; 79.234ºW | 10.98 | 3.4 |
| [MSB:BIRD:28213](http://arctos.database.museum/guid/MSB:Bird:28213) | *tyrianthina* | Lambayeque; | 6.237ºS; 79.234ºW | 11.18 | 3.51 |
| [MSB:BIRD:28214](http://arctos.database.museum/guid/MSB:Bird:28214) | *tyrianthina* | Lambayeque; | 6.237ºS; 79.234ºW | 11.08 | 3.33 |
| [MSB:BIRD:28216](http://arctos.database.museum/guid/MSB:Bird:28216) | *tyrianthina* | Lambayeque; | 6.237ºS; 79.234ºW | 9.92 | 3.95 |
| [MSB:BIRD:28218](http://arctos.database.museum/guid/MSB:Bird:28218) | *tyrianthina* | Lambayeque; | 6.237ºS; 79.234ºW | 11.18 | 4 |
| [MSB:BIRD:28227](http://arctos.database.museum/guid/MSB:Bird:28227) | *tyrianthina* | Lambayeque; | 6.237ºS; 79.234ºW | 10.14 | 3.39 |
| [MSB:BIRD:28236](http://arctos.database.museum/guid/MSB:Bird:28236) | *tyrianthina* | Lambayeque; | 6.237ºS; 79.234ºW | 11.46 | 3.76 |
| [MSB:BIRD:28237](http://arctos.database.museum/guid/MSB:Bird:28237) | *tyrianthina* | Lambayeque; | 6.237ºS; 79.234ºW | 10.00 | 3.5 |
| [MSB:BIRD:28241](http://arctos.database.museum/guid/MSB:Bird:28241) | *tyrianthina* | Lambayeque; | 6.237ºS; 79.234ºW | 11.10 | 3.99 |
| [MSB:BIRD:28028](http://arctos.database.museum/guid/MSB:Bird:28028) | *tyrianthina* | Lambayeque; Dos Lagunas | 6.237ºS; 79.234ºW | 11.59 | 3.5 |
| [MSB:BIRD:28061](http://arctos.database.museum/guid/MSB:Bird:28061)* | *tyrianthina* | Lambayeque; Tres Lagunas | 6.237ºS; 79.234ºW | 13.24 |  |
| [MSB:BIRD:31314](http://arctos.database.museum/guid/MSB:Bird:31314)* | *tyrianthina* | Lambayeque; Tres Lagunas | 6.237ºS; 79.234ºW | 11.28 | 4.23 |
| [MSB:BIRD:31315](http://arctos.database.museum/guid/MSB:Bird:31315)* | *tyrianthina* | Lambayeque; Tres Lagunas | 6.237ºS; 79.234ºW | 12.44 | 3.56 |
| [MSB:BIRD:31321](http://arctos.database.museum/guid/MSB:Bird:31321)* | *tyrianthina* | Lambayeque; Tres Lagunas | 6.237ºS; 79.234ºW |  |  |
| [MSB:BIRD:31323](http://arctos.database.museum/guid/MSB:Bird:31323)* | *tyrianthina* | Lambayeque; Tres Lagunas | 6.237ºS; 79.234ºW | 10.96 | 3.3 |
| [MSB:BIRD:31326](http://arctos.database.museum/guid/MSB:Bird:31326)* | *tyrianthina* | Lambayeque; Tres Lagunas | 6.237ºS; 79.234ºW | 11.45 | 3.79 |
| [MSB:BIRD:31328](http://arctos.database.museum/guid/MSB:Bird:31328)* | *tyrianthina* | Lambayeque; Tres Lagunas | 6.237ºS; 79.234ºW | 10.48 | 3.52 |
| [MSB:BIRD:31344](http://arctos.database.museum/guid/MSB:Bird:31344)* | *tyrianthina* | Lambayeque; Tres Lagunas | 6.237ºS; 79.234ºW | 9.98 | 3.41 |
| [MSB:BIRD:31348](http://arctos.database.museum/guid/MSB:Bird:31348)* | *tyrianthina* | Lambayeque; Tres Lagunas | 6.237ºS; 79.234ºW | 11.92 | 3.93 |
| [MSB:BIRD:31350](http://arctos.database.museum/guid/MSB:Bird:31350)* | *tyrianthina* | Lambayeque; Tres Lagunas | 6.237ºS; 79.234ºW | 9.05 | 3.3 |
| [MSB:BIRD:31351](http://arctos.database.museum/guid/MSB:Bird:31351)* | *tyrianthina* | Lambayeque; Tres Lagunas | 6.237ºS; 79.234ºW | 12.1 | 3.5 |
| [MSB:BIRD:31353](http://arctos.database.museum/guid/MSB:Bird:31353)* | *tyrianthina* | Lambayeque; Tres Lagunas | 6.237ºS; 79.234ºW | 11.66 | 3.7 |
| [MSB:BIRD:31357](http://arctos.database.museum/guid/MSB:Bird:31357)* | *tyrianthina* | Lambayeque; Tres Lagunas | 6.237ºS; 79.234ºW |  |  |
| [MSB:BIRD:31359](http://arctos.database.museum/guid/MSB:Bird:31359)* | *tyrianthina* | Lambayeque; Tres Lagunas | 6.237ºS; 79.234ºW |  |  |
| [MSB:BIRD:31325](http://arctos.database.museum/guid/MSB:Bird:31325)* | *tyrianthina* | Lambayeque; Tres Lagunas | 6.237ºS; 79.234ºW |  |  |
| [MSB:BIRD:31356](http://arctos.database.museum/guid/MSB:Bird:31356) | *tyrianthina* | Lambayeque; Tres Lagunas | 6.237ºS; 79.234ºW | 11.04 | 3.86 |
| [MSB:BIRD:31364](http://arctos.database.museum/guid/MSB:Bird:31364) | *tyrianthina* | Lambayeque; Tres Lagunas | 6.237ºS; 79.234ºW | 11.32 | 3.7 |
| [MSB:BIRD:31365](http://arctos.database.museum/guid/MSB:Bird:31365) | *tyrianthina* | Lambayeque; Tres Lagunas | 6.237ºS; 79.234ºW | 11.16 | 3.87 |
| [MSB:BIRD:31368](http://arctos.database.museum/guid/MSB:Bird:31368) | *tyrianthina* | Lambayeque; Tres Lagunas | 6.237ºS; 79.234ºW | 11.12 | 3.33 |
| [MSB:BIRD:31370](http://arctos.database.museum/guid/MSB:Bird:31370) | *tyrianthina* | Lambayeque; Tres Lagunas | 6.237ºS; 79.234ºW | 12.43 | 3.6 |
| [MSB:BIRD:31371](http://arctos.database.museum/guid/MSB:Bird:31371) | *tyrianthina* | Lambayeque; Tres Lagunas | 6.237ºS; 79.234ºW | 11.6 | 3.78 |
| [MSB:BIRD:31372](http://arctos.database.museum/guid/MSB:Bird:31372) | *tyrianthina* | Lambayeque; Tres Lagunas | 6.237ºS; 79.234ºW | 11.42 | 3.54 |
| [MSB:BIRD:31374](http://arctos.database.museum/guid/MSB:Bird:31374) | *tyrianthina* | Lambayeque; Tres Lagunas | 6.237ºS; 79.234ºW | 12.19 | 3.97 |
| [MSB:BIRD:31376](http://arctos.database.museum/guid/MSB:Bird:31376) | *tyrianthina* | Lambayeque; Tres Lagunas | 6.237ºS; 79.234ºW | 11.81 | 3.72 |
| [MSB:BIRD:31380](http://arctos.database.museum/guid/MSB:Bird:31380) | *tyrianthina* | Lambayeque; Tres Lagunas | 6.237ºS; 79.234ºW | 11.9 | 2.5 |
| [MSB:BIRD:31381](http://arctos.database.museum/guid/MSB:Bird:31381) | *tyrianthina* | Lambayeque; Tres Lagunas | 6.237ºS; 79.234ºW | 10.82 | 3.64 |
| [MSB:BIRD:31383](http://arctos.database.museum/guid/MSB:Bird:31383) | *tyrianthina* | Lambayeque; Tres Lagunas | 6.237ºS; 79.234ºW | 11.26 | 3.67 |
| [MSB:BIRD:31385](http://arctos.database.museum/guid/MSB:Bird:31385) | *tyrianthina* | Lambayeque; Tres Lagunas | 6.237ºS; 79.234ºW | 11.89 | 3.91 |
| LSUMZ-78123 | *tyrianthina* | Piura; 33km (by road) SW Huancabamba | 5.331ºS; 79.359ºW | 10.72 | 3 |
| LSUMZ-78122 | *tyrianthina* | Piura; 35km (by road) SW Huancabamba | 5.331ºS; 79.359ºW | 10.42 | 3 |

**Table S2:** Loadings from principal component analysis of temperature, precipitation, and seasonality Bioclim variables.

| **Temperature** | **Comp.1** | **Comp.2** | **Comp.3** | **Comp.4** |
| --- | --- | --- | --- | --- |
| Annual Mean Temperature | -0.385 | -0.15 | 0.56 | -0.706 |
| Isothermality (2/7) (* 100) | -0.147 | 0.825 | 0.506 | -0.174 |
| Max Temp Warmest Month | -0.344 | -0.398 | 0.439 | -0.431 |
| Min Temp Coldest Month | -0.357 | 0.321 | -0.734 | -0.141 |
| Mean Temp Wettest Quarter | -0.381 | -0.116 | 0.728 | 0.538 |
| Mean Temp Driest Quarter | -0.385 | -0.266 | 0.424 | -0.696 |
| Mean Temp Warmest Quarter | -0.378 | -0.174 | 0.309 | -0.117 |
| Mean Temp Coldest Quarter | -0.385 | -0.305 | 0.534 | 0.403 |
| Proportion of Variance: | 0.8373 | 0.1555 | 0.0034 | 0.0026 |
| Cumulative proportion: | 0.8373 | 0.9928 | 0.9963 | 0.9989 |

| **Precipitation** | **Comp.1** | **Comp.2** | **Comp.3** | **Comp.4** |
| --- | --- | --- | --- | --- |
| Annual Precipitation | -0.417 | 0.107 | -0.309 | -0.494 |
| Precip Wettest Month | -0.353 | 0.474 | 0.193 | 0.716 |
| Precip Driest Month | -0.378 | -0.412 | 0.149 | -0.811 |
| Precip of Wettest Quarter | -0.333 | 0.518 | -0.508 | -0.107 |
| Precip Driest Quarter | -0.383 | -0.394 | 0.146 | 0.334 |
| Precip Warmest Quarter | -0.388 | 0.175 | 0.77 | -0.421 |
| Precip Coldest Quarter | -0.387 | -0.375 | -0.12 | 0.102 |
| Proportion of Variance: | 0.7801 | 0.1795 | 0.0307 | 0.0076 |
| Cumulative proportion: | 0.7801 | 0.9596 | 0.9904 | 0.9980 |

| **Seasonality** | **Comp.1** | **Comp.2** | **Comp.3** | **Comp.4** |
| --- | --- | --- | --- | --- |
| Mean Monthly Temp Range | -0.517 | -0.223 | 0.619 | 0.548 |
| Temp Seasonality (STD * 100) | -0.504 | -0.187 | -0.777 | 0.326 |
| Temp Annual Range | -0.531 | -0.35 | 0.108 | -0.764 |
| Precipitation Seasonality (CV) | -0.444 | 0.891 |  |  |
| Proportion of Variance: | 0.8293 | 0.1093 | 0.0578 | 0.0037 |
| Cumulative proportion: | 0.8293 | 0.9386 | 0.9963 | 1 |

**Table S3:** Loadings from principal component analysis of all 19

Bioclim variables.

| **Bioclim Variables** | **Comp. 1** | **Comp. 2** | **Comp. 3** | **Comp. 4** |
| --- | --- | --- | --- | --- |
| Annual Mean Temperature | 0.000 | 0.007 | -0.024 | 0.007 |
| Mean Diurnal Range | -0.002 | -0.007 | -0.013 | 0.017 |
| Isothermality | 0.002 | 0.058 | 0.036 | 0.007 |
| Temperature seasonality | -0.030 | -0.353 | -0.113 | -0.675 |
| Max Temp Warmest Month | -0.001 | 0.000 | -0.037 | 0.015 |
| Min Temp Coldest Month | 0.001 | 0.021 | -0.013 | -0.002 |
| Temp Annual Range | -0.003 | -0.022 | -0.024 | 0.017 |
| Mean Temp Wettest Quarter | -0.001 | 0.005 | -0.020 | 0.001 |
| Mean Temp Driest Quarter | 0.000 | 0.012 | -0.022 | 0.017 |
| Mean Temp Warmest Quarter | -0.001 | 0.004 | -0.025 | 0.000 |
| Mean Temp Coldest Quarter | 0.000 | 0.013 | -0.022 | 0.017 |
| Annual Precipitation | 0.907 | 0.058 | -0.269 | 0.127 |
| Precip Wettest Month | 0.107 | -0.164 | 0.207 | -0.157 |
| Precip Driest Month | 0.031 | 0.145 | 0.012 | -0.145 |
| Precip Seasonality | -0.058 | -0.183 | 0.221 | -0.222 |
| Precip Wettest Quarter | 0.286 | -0.633 | 0.076 | -0.146 |
| Precip Driest Quarter | 0.112 | 0.441 | 0.022 | -0.456 |
| Precip Warmest Quarter | 0.230 | 0.094 | 0.900 | 0.072 |
| Precip Coldest Quarter | 0.114 | 0.427 | 0.002 | -0.444 |
| Proportion of Variance: | 0.9313 | 0.0455 | 0.0174 | 0.0032 |
| Cumulative proportion: | 0.9313 | 0.9768 | 0.9942 | 0.9974 |

**Table S4:** Parameter settings for each of the four IMa2 analyses.

| **Transect** | **divergence time (-t)** | **migration (-m)** | **theta (-q)** | **genealogies sampled** |
| --- | --- | --- | --- | --- |
| Vilcanota | 3 | 12 | 4 | 100 000 |
| Manu Road | 3 | 150 | 4 | 250 000 |
| Control- Dry | 3 | 50 | 4 | 200 000 |
| Control- Wet | 3 | 100 | 6 | 250 000 |

**Table S5**: Among population comparisons based on 845bp ND2 data. Below diagonal: percent corrected pairwise differences among all populations. Above diagonal: Fst values among

populations. All values were significant.

|  | A | B | C | D | E | F |
| --- | --- | --- | --- | --- | --- | --- |
| Clade A | - | 0.91 | 0.85 | 0.89 | 0.90 | 0.83 |
| Clade B | 2.39 | - | 0.73 | 0.92 | 0.92 | 0.99 |
| Clade C | 2.02 | 0.24 | - | 0.79 | 0.85 | 0.77 |
| Clade D | 2.13 | 0.9 | 0.43 | - | 0.88 | 0.86 |
| Clade E | 2.41 | 1.24 | 0.85 | 0.91 | - | 0.85 |
| Clade F | 2.03 | 1.07 | 0.67 | 0.79 | 1.04 | - |

**Table S6:** Results from AMOVA analyses. The six assigned groups correspond to the six clades in the phylogeny (Fig. 2a) and are divided by putative topographic barriers highlighted in Fig. 2c.

| **Source of Variation** | **d.f.** | **Sum of Squares** | **Variance Components** | **Percentage Variation** |
| --- | --- | --- | --- | --- |
| Among Groups | 5 | 732.98 | 6.78 | 87.95 |
| Among Populations within groups | 10 | 22.63 | 0.21 | 2.68 |
| Within Populations | 120 | 86.66 | 0.72 | 9.36 |

**Table S7:** Standard indices of molecular diversity for each clade based on 845bp ND2. Sample size (n), number of variable sites (var), nucleotide diversity (π), haplotype diversity (Hd), Fu's F and Tajima's D are reported. Significant results for Fu's F and Tajima's D suggest deviations from neutrality.

| **Population** | **n** | **Var** | **π** | **Hd** | **Fu's F** | **Tajima's D** |
| --- | --- | --- | --- | --- | --- | --- |
| Clade A | 30 | 27 | 0.003859 | 0.770 | 0.49 | -1.8 * |
| Clade B | 21 | 2 | 0.000093 | 0.095 | -0.92 ** | -1.16^ns^ |
| Clade C | 12 | 6 | 0.002239 | 0.682 | -1.15^ns^ | -1.94** |
| Clade D | 30 | 12 | 0.001202 | 0.747 | -4.86 *** | -2.08 ** |
| Clade E | 41 | 19 | 0.001724 | 0.827 | 1.09 | -1.29^ns^ |
| Clade F | 2 | 1 | 0.001103 | 0.5 | 0^ns^ | 0^ns^ |

^ns^ p>0.05; * p <0.05; ** p <0.01; *** p<0.001
